# Fei-Yan-Qing-Hua decoction attenuates influenza virus infection by enhancing host antiviral response through microbiota-derived acetate

**DOI:** 10.3389/fphar.2024.1446749

**Published:** 2024-10-10

**Authors:** Biao Dou, Xiao Wu, Yurong He, Guihua Xu, Huan Zhang, Qilin Huang, Xuan Chen, Naifan Duan, Linqiong Zhou, Wei Zhang, Huazhang An, Yuejuan Zheng

**Affiliations:** ^1^ The Research Center for Traditional Chinese Medicine, Shanghai Institute of Infectious Diseases and Biosecurity, Shanghai University of Traditional Chinese Medicine, Shanghai, China; ^2^ Center for Traditional Chinese Medicine and Immunology Research, School of Integrative Medicine, Shanghai University of Traditional Chinese Medicine, Shanghai, China; ^3^ Department of Medical Laboratory, The First Affiliated Hospital of Henan University, Kaifeng, China; ^4^ Department of Pulmonary Diseases, ShuGuang Hospital Affiliated to Shanghai University of Traditional Chinese Medicine, Shanghai, China

**Keywords:** influenza virus, Fei-Yan-Qing-Hua decoction, interferon β, gut microbiota, short-chain fatty acids (SCFAs)

## Abstract

**Background:**

Fei-Yan-Qing-Hua decoction (FYQHD) is derived from the well-known Ma Xing Shi Gan decoction, which was documented in Zhang Zhong Jing’s “Treatise on Exogenous Febrile Disease” during the Han Dynasty. Although FYQHD has been used in the treatment of pneumonia and has demonstrated clinical efficacy for decades, the underlying mechanism by which FYQHD protects against influenza virus infection through modulation of gut flora remains unclear. Here, we examined the regulatory impacts of FYQHD on an influenza virus-infected mouse model and explored the mechanisms involved.

**Methods:**

An infectious mouse model was created by intranasal instillation of influenza A virus (IAV). The effectiveness of FYQHD was assessed through various measures, including weight loss, lung wet/dry ratio, oxidative stress levels, viral load in lung tissues, and intestinal injuries. Changes in gut microbiota and SCFA production were also examined.

**Results:**

The results showed that FYQHD significantly reduced viral load, increased the production of type I interferon (IFN-I), and restored the integrity of the intestinal barrier following IAV challenge. Additionally, FYQHD significantly corrected the dysbiosis of gut microbiota induced by influenza virus infection, enhancing the abundance of SCFA-producing bacteria and acetate production. However, the depletion of gut microbiota significantly attenuated the protective effects of FYQHD against influenza virus infection. *In vitro*, the antiviral effect of acetate was demonstrated through the upregulation of concentrations of IFN-β.

**Conclusion:**

FYQHD attenuates influenza virus-induced lung and intestinal injuries by boosting the host antiviral response through increasing the abundance of *Lachnospiraceae_NK4A136* and *Roseburia,* along with elevated acetate levels. The study advances our understanding of the therapeutic mechanisms of FYQHD and provides a theoretical basis for the application of FYQHD in the treatment of influenza.

## 1 Introduction

The emergence of coronavirus disease (COVID-19) caused by severe acute respiratory syndrome coronavirus 2 (SARS-CoV-2) has garnered global attention since its initial report in December 2019 (Zhu et al., 2020). However, the global burden of influenza, especially that caused by influenza A virus (IAV), remains a critical public health concern. Influenza viruses are crucial respiratory pathogens that cause substantial seasonal outbreaks and pandemics, leading to high morbidity and mortality worldwide (Doherty et al., 2006; Herold et al., 2015). Particularly in recent years, seasonal influenza outbreaks and global pandemics happen frequently. Approximately 10%–30% of the global population is affected by influenza virus infection every year, resulting in about 3–5 million severe cases and up to 290,000–650,000 deaths from acute lung injury (ALI) or acute respiratory distress syndrome (ARDS) (Iuliano et al., 2018; Waehre et al., 2022). Vaccination is the main strategy for preventing influenza A. However, the high susceptibility of IAV to antigenic drift and shift and delays in vaccine production significantly challenge its effectiveness. Presently, a limited number of drugs can target the influenza M2 ion channel protein, whereas several inhibitors of influenza NA, RNA-dependent RNA polymerase, and protease, such as oseltamivir (OSV), baloxavir and favipiravir, etc., are either approved for clinical use or under investigation (Zhang et al., 2020). Oseltamivir is a well-known antiviral effective against influenza. However, due to its neuraminidase inhibition mechanism, its protective effect is temporary and requires administration within 48 h of infection to significantly limit viral replication and relieve symptoms. In summary, evolving mutations in IAV and resulting drug resistance challenge influenza treatment, underscoring the urgent need for new antiviral agents.

Traditional Chinese medicine (TCM) plays a significant role in treating viral pneumonia caused by SARS and SARS-CoV-2 due to its proven effectiveness and lower incidence of side effects (Huang et al., 2021). Chinese herbal formulas have unique medicinal effects resulting from the combination of various herbal medicines, exhibiting anti-infective effects through multiple mechanisms, such as antiviral, anti-inflammatory, and immunomodulatory effects (Xi et al., 2020). Fei-Yan-Qing-Hua decoction (FYQHD), derived from Ma-Xing-Shi-Gan decoction and consisting of five herbs known for dispersing phlegm and relieving fever or asthma. FYQHD had demonstrated efficacy against community-acquired pneumonia (CAP) in the clinic for 2 decades at Shuguang Hospital, affiliated with Shanghai University of Traditional Chinese Medicine. The multiple compounds in FYQHD can impact various aspects of the influenza virus, ensuring its effectiveness against influenza virus and its mutants.

The gut microbiome is crucial in the shift from homeostasis to disease. Studies have revealed a bi-directional link between the lungs and the gut, termed the lung-gut axis (He et al., 2017). Furthermore, according to TCM theory, “the lung and the large intestine are interior-exterior,” suggesting a close relationship between lung diseases and the function of the large intestine. Literatures reported that IAV infection in mice is often linked to intestinal damage, including increased permeability and reduced levels of the zonula occludens-1 (ZO-1) protein (Zhu et al., 2018). Meanwhile, the gut microbiota also plays an important role in protecting the host from the infection of influenza virus (Stefan et al., 2020). According to TCM theory, dietary supplements with non-digestible carbohydrates, antioxidant phytochemicals, and natural compounds can pass through the stomach to the intestine, where gut microbiota can transform or ferment them into beneficial metabolites. Therefore, TCM components that regulate the gut-lung axis seem to be a promising strategy to alleviate IAV-induced lung and gut immune injury.

Gut bacteria partially break down non-digestible polysaccharides to produce short-chain fatty acids (SCFAs) (Rudiansyah et al., 2022). Among these SCFAs, acetate, propionate, and butyrate are well-documented for their crucial roles in regulating the host immune response (Kim, 2021). The activation of various interferon-stimulated genes (ISGs) by type I interferons is a key antiviral defense mechanism (Ivashkiv and Donlin, 2014). Notably, acetate has the capacity to modulate the host’s immune response to viral infections. By activating G-protein coupled receptor 43 (GPR43), acetate induces an IFN-I response in lung epithelial cells, thereby suppressing the replication of respiratory syncytial virus (RSV) (Antunes et al., 2019). Furthermore, recent studies have demonstrated that the probiotic *Bifidobacterium pseudolongum NjM1* exhibits protective properties against IAV infection. This protective effect is associated with acetate production, which enhances MAVS clustering to activate IRF3, thus promoting the expression of IFN-I (Niu et al., 2023). In addition, IAV infection triggers the expression of superoxide dismutase (SOD) and xanthine oxidase, leading to a decrease in antioxidant levels and the onset of oxidative stress (OS) (Peterhans, 1997). Studies have shown that acetate and butyrate exhibit potent antioxidative effects at a concentration of 1 mM (Hu S. et al., 2020).

Although FYQHD has been used clinically for 2 decades, its precise mechanism of protecting against influenza, especially regarding its impact on gut function, remains unclear. FYQHD treatment was found to relieve lung damage and oxidative stress in flu mice, while altering the diversity and structure of gut microbiota to resemble that of normal mice. Additionally, it increased the abundance of SCFA-producing bacteria like *Roseburia, Clostridium, and Lachnospiraceae* (Sun et al., 2020; Zhao et al., 2021). The study also investigated the effect of FYQHD on mice infected with IAV by depleting gut microbiota. Finally, the protective effect of FYQHD was linked to its ability to enhance the gut microbiota-derived acetate, which in turn boosted the induction of IFN-I during IAV infection. The current results provide evidence that FYQHD has therapeutic potential for the treatment of IAV infection. Furthermore, it offers insights into the mechanisms by which it influences the host and gut microbiota, laying the groundwork for understanding its clinical effectiveness.

## 2 Materials and methods

### 2.1 Reagents and chemicals

All traditional Chinese medicine materials were provided by the Department of Pulmonary Diseases, ShuGuang Hospital affiliated to Shanghai University of Traditional Chinese Medicine. Influenza virus A/Puerto Rico/8/1934 H1N1 (PR8) was used *in vitro* and *in vivo* experiments. Oseltamivir phosphate granules (OSV) was purchased from Yichang HEC Changjiang Pharmaceutical Co., Ltd. (Hubei, China). All the antibiotics used in this study were purchased from Sangon Biotech (Shanghai) Co.

### 2.2 Preparation and analysis of FYQHD for quality control

The FYQHD decoction was prepared following the methodology outlined in our previous report (Zhang et al., 2024). FYQHD consists of *Ephedra dahurica* Turcz. (Ephedraceae) (6 g), *Prunus mandshurica* (Maxim.) Koehne. (Rosaceae) (9 g), *Gypsum Fibrosum* (Sulfates) (30 g), *Glycyrrhiza uralensis* Fisch. ex DC. (Fabaceae) (9 g), *Bupleurum chinensis* DC. (Apiaceae) (9 g), *Scutellaria baicalensis* Georgi (Lamiaceae) (9 g), *Rheum officinale* Baill. (Polygonaceae) (6 g), *Fagopyrum acutatum* Mansf. ex K. Hammer (Polygonaceae) (30 g) and *Morus alba var. tatarica* (L.) Loudon (Moraceae) (9 g) in a ratio of 1:1.5:5:1.5:1.5:1.5:1:5:1.5. Detailed information on FYQHD is provided in Supplementary Table S1. While the family names, documented bioactivities, and identified compounds of these herbal remedies are elaborated in our prior investigation (Zhang et al., 2024).

A mixture of one mineral drug and 8 botanical drugs was soaked for 30 min with 5-fold of purified water (volume/weight), then decocted for 1 h. After completing the first decoction, the liquid was poured out and the remaining mixture was decocted for the second time by adding twice the volume of purified water. Once the second decoction was finished, the two decoctions were mixed and subjected to filter. Eventually, the filtered liquid was concentrated to 3.2 g crude drug/ml. FYQHD decoctions were freshly preserved at −20°C for no longer than 2 days prior to their application.

The chromatographic fingerprint of FYQHD was performed using UPLC-MS/MS. Then, 17 metabolites are confirmed by authentic standards, which are the major metabolites of corresponding botanical drugs in FYQHD (Committee, 2020), including ephedrine and pseudoephedrine from *Ephedra dahurica* Turcz., amygdalinare from *Prunus mandshurica* (Maxim.) Koehne., epicatechin and isorhamnetin from *Fagopyrum acutatum* Mansf. ex K. Hammer, baicalin, wogonin, luteolin and wogonoside from *Scutellaria baicalensis* Georgi, liquiritin and glycyrrhizic acid from *Glycyrrhiza uralensis* Fisch. ex DC., rhein and emodin from *Rheum officinale* Baill., morusin and kaempferol from *Morus alba var*. *tatarica* (L.) Loudon, saikosaponin D and saikosaponin A from *Bupleurum chinensis* DC. Typical base peak and ion flow chromatograms are shown in Supplementary Figure S1. And the standard curve of standards used in UPLC-MS/MS are shown in Supplementary Figure S2. Detailed characterizations for each metabolite are provided in Supplementary Table S2.

### 2.3 Lung index indicators

At specified time points following infection, mice were euthanized in accordance with approved protocols. The lungs were meticulously excised and rinsed with physiological saline solution. Subsequently, the total lung weight was recorded to calculate the lung index using the following formula: $\text{lung index}=\left(\text{lung wet weight}/\text{body weight}\right)\times 100\%$ .

### 2.4 Animals

Female C57BL/6J mice (7 weeks old, 17–20 g) were obtained from Bikai Laboratory Animals Ltd. and then housed in a standard animal room (room temperature: 22°C ± 2°C; relative humidity: 30%–40%; light conditions: 12 h dark/light cycle).

Mice were randomly divided into 7 groups: control group, PR8 group, PR8 + OSV (30 mg/kg) group, PR8 + FYQHD (L) (low dosage, 24 g/kg) group, PR8 + FYQHD (M) (medium dosage, 48 g/kg) group, PR8 + FYQHD (H) (high dosage, 96 g/kg) group. In addition, the low dose administered to the mice corresponds to the dose used clinically in patients. Each mouse, weighing 20 g, received an administration of 150 µL FYQHD at a concentration of 3.2 g/mL. All groups of mice excepted control group were infected intranasally by influenza virus PR8 (2 × 10^4^ PFU in 50 μl/mouse) intranasally and were then administered the indicated drugs within 2 h after infection. For the survival observation for 14 days, mice were monitored daily for body weight loss. If a mouse loses body weight over 25% of its original body weight, it would be humanely euthanized immediately.

Antibiotic Treatment: Mice were treated with a broad-spectrum cocktail of antibiotics to deplete the gut microbiota, as previously described (Ichinohe et al., 2011). The antibiotic cocktail (ABX) comprised 4 antibiotics: ampicillin (1 g/L), vancomycin (0.5 g/L), neomycin (1 g/L), and metronidazole (1 g/L). The feeding water containing antibiotics was changed every 2 days. After 28 days, mice were infected with the same volume of PR8 intranasally and were then administered the indicated drugs within 2 h after infection.

### 2.5 The detection of H1N1 viral titers

Mice were randomly divided into 2 groups (n = 6) and intranasally infected by PR8. Subsequently, the mice received a low dose of FYQHD (150 μL/day/mouse) for 2 or 3 days post-infection (dpi). Lung samples were homogenized and centrifuged at 13,000 rpm for 1 min at 4°C. The lung homogenate supernatant was transferred to a 96-well plate containing MDCK cells using a serial 10-fold dilution method. The cells were then incubated at 37°C in a 5% CO_2_ environment to assess cytopathic effects (CPE). The TCID_50_ was determined using the Reed-Muench method and reported as Log_10_ TCID_50_/0.1 mL.

### 2.6 16S rRNA gene sequencing and analysis

Six cecum samples were collected and frozen at −80°C until 16S rRNA sequencing from each group on the 7th day after PR8 infection. Total genomic DNA was purified with a MagPure Soil DNA LQ Kit following the manufacturer’s instructions. DNA concentration was determined with NanoDrop 2000 (Thermo Fisher Scientific, United States) and DNA integrity was evaluated by agarose gel electrophoresis. DNA samples were used as the template to PCR-amplify bacterial 16S rRNA genes with barcoded primers and a Taq polymerase (Takara, Dalian, China). For bacterial diversity analysis, V3-V4 variable regions of 16S rRNA genes were amplified with universal primers 343F (5′-TACGGRAGGCAGCAG-3′) and 798R (5′-AGG​GTA​TCT​AAT​CCT-3′) (Nossa et al., 2010). The PCR products were analyzed by agarose gel electrophoresis, purified, and quantified using Qubit dsDNA Assay Kit (Thermo Fisher Scientific, United States). The 16S rRNA gene sequencing was conducted by Shanghai OE Biotech. Co., Ltd. (Shanghai, China) following the established method (Ye et al., 2021).

### 2.7 SCFA concentration analysis

The levels of SCFAs in the sera of mice were measured with GC–MS analysis. Serum samples (400 μL) were supplemented with 100 μL 50% sulfuric acid. Then 500 μL of the supernatants was mixed with 500 μL of ethyl ether, vortexed (2 min) and centrifuged (12,000 × g, 10 min). The extracted samples underwent separation using an Agilent DB-WAX capillary column (30 m × 0.25 mm ID × 0.25 µm) gas chromatography system and detection was carried out using the 7000B GC-MS system by Agilent (United States). Mass Hunter Workstation software was used to quantitative analysis. The concentrations of SCFAs were determined based on standard curves.

### 2.8 Total RNA extraction and quantitative reverse-transcriptase PCR

Trizol reagent (Invitrogen, United States) was used for isolating total RNA from lung tissues of mice and RNA extraction kit (RNAfast200, Fastagen, Shanghai, China) was used for cells. 2^−△△CT^ method was performed to calculate the expression of relative mRNA in this study with β-Actin as a normalized reference. The primer sequences were listed in Supplementary Table S3.

### 2.9 Assessment of antioxidant indexes in the lungs and sera

Frozen lung tissues were homogenized with RIPA lysis buffer to extract total proteins. Following the centrifugation of 12,000 g for 15 min at 4°C, the protein content was measured using a bicinchoninic acid (BCA) protein assay kit as per the manufacturer’s guidelines. Quantification of key redox enzymes such as MPO, T-SOD, CAT, T-AOC, GSH-Px, and MDA was performed using commercial kits obtained from Nanjing Jiancheng Bioengineering Institute, Nanjing, China, with adherence to the manufacturer’s protocols.

### 2.10 *In vitro* sodium acetate treatment

A549 cells were pretreated with 260 μM sodium acetate (S5636, SIGMA) for 24 h in DMEM containing 10% FBS and the cells were infected with PR8 (MOI = 2) for 8 h and 24 h, as described previously (Niu et al., 2023). Viral loads were measured by Quantitative Real-time Polymerase Chain Reaction Analysis.

### 2.11 Histopathological examination

Freshly harvested lung tissues and proximal colon tissues of mice were fixed in 4% formaldehyde and paraffin-embedded, and then cut into 3 μm-thick sections for hematoxylin-eosin (HE) staining. These sections were further captured by light microscopy using an Axio Imager M2 microscope (Carl Zeiss Micro Imaging, Gottingen, Germany).

### 2.12 Immunohistochemistry

Immunohistochemical analysis was performed to assess the expression levels of zonula occludens 1 (ZO-1), Claudin-1, and Occludin, following established protocols (Schroeder et al., 2018). Briefly, colonic tissue slides embedded in paraffin were deparaffinized, treated with a citrate buffer solution (0.01 M, pH 6.0), and subjected to autoclave sterilization at 120°C for 5 min to aid in antigen retrieval. Following rinsing, incubation at room temperature for 60 min ensued after the addition of 5% bovine serum albumin (BSA). Without any preceding wash steps, rabbit anti-mouse primary antibodies against ZO-1, Claudin-1 and Occludin were added dropwise respectively, and the samples were then incubated overnight at 4°C. Tissue slides were washed in PBS and then exposed to a fluorophore-conjugated secondary antibody (diluted 1:200 in block solution) for 1 h. After the final wash, the sample was treated with 3,3′-diaminobenzidine tetrahydrochloride (DAB) at room temperature for 60 min.

### 2.13 Alcian blue staining

In the process of Alcian blue staining, the sections were dried in an oven and heated at 66°C for 20–30 min. Subsequently, 3 rounds of xylene and 3 rounds of ethanol were sequentially applied. Each slice was treated with 100 μL of Alcian blue staining solution, followed by incubation in a humid chamber for 1 h. The staining solution was aspirated, and 100 μL of nuclear solid red staining solution was introduced to each slice, with subsequent washing in water. The samples were then subjected to three sequential treatments of ethanol, phenol-xylene I, and xylene II.

### 2.14 Statistical analysis

The data were analyzed with GraphPad Prism version 8.0, and the results were displayed as mean ± SD. Variations in mean values between groups were evaluated using an unpaired two-tailed Student’s *t*-test. Statistical comparison of parameters across the 3 groups involved one-way analysis of variance (ANOVA) followed by Tukey’s test. Correlation analysis was carried out using the Pearson statistical method. A *P* value < 0.05 was deemed to indicate statistical significance.

## 3 Results

### 3.1 Quantitative analysis of the major components in FYQHD

FYQHD is composed of 9 traditional Chinese medicines, and detailed information on FYQHD is provided in Supplementary Table S1. In addition, UPLC-MS/MS was utilized to establish the quality control of FYQHD and to quantify its representative metabolites. The total positive and negative ion chromatograms of the samples revealed the primary bioactive metabolites present in FYQHD-medicated serum (Supplementary Figure S1). Subsequently, 17 metabolites were verified using authentic standards, representing the primary metabolites of the respective botanical drugs in FYQHD (Committee, 2020) (Supplementary Figure S2). Comprehensive characterizations for each metabolite were presented in Supplementary Table S2.

### 3.2 FYQHD protected mice from lung injury induced by IAV infection

**FIGURE 1:**
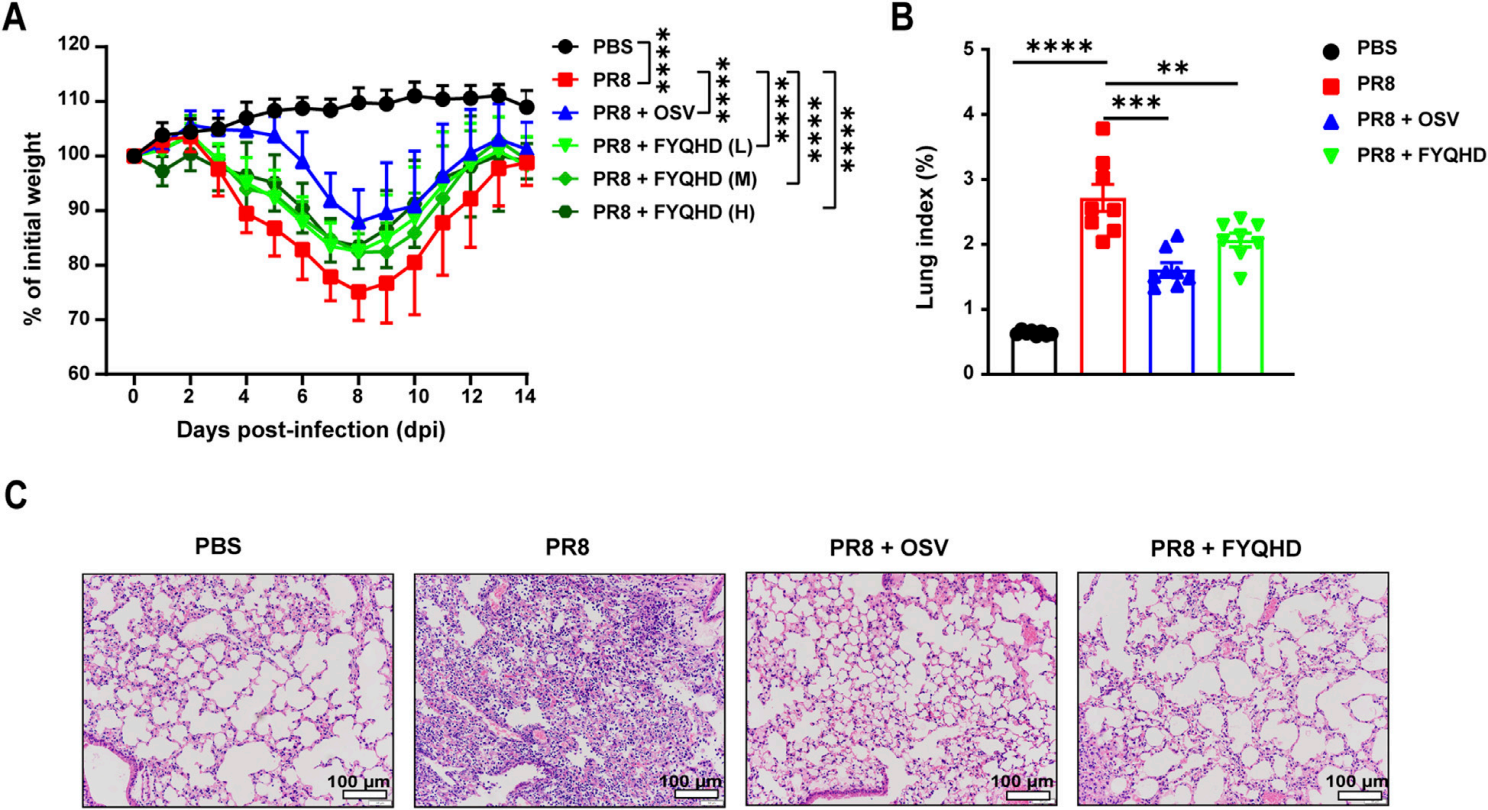
FYQHD attenuated the body weight loss and alleviated lung damage during IAV infection. Mice were infected with 50 µL PBS containing influenza A/PuertoRico/8/1934 H1N1 (PR8) virus, and were orally administered with FYQHD or Oseltamivir within 2 h after infection. **(A)** The percentage of weight loss post infection relative to initial body weight (day 0) (n = 14). PBS: Normal group, PR8: IAV group, PR8 + FYQHD (L): FYQHD-treated low-dose group, 24 g/kg, PR8 + FYQHD (M): FYQHD-treated medium-dose group, 48 g/kg, PR8 + FYQHD (H): FYQHD-treated high-dose group, 96 g/kg, PR8 + OSV: Oseltamivir-treated group, 30 mg/kg. **(B)** The change of lung indices of mice in each group (n = 8). **(C)** The pathological observation of mouse lung tissues. The dose of FYQHD used in **(B, C)** was 24 g/kg. Data were presented as mean ± SEM for each group. *P < 0.05, **P < 0.01, ***P < 0.001, ****P < 0.0001 vs. PR8 group. Scale bars represent 100 μm.

The therapeutic characteristic of FYQHD was assessed using the viral infection mouse model. Oseltamivir was used as an effective treatment. The mice’s weights started to noticeably decrease on day 4 post-infection, reached their lowest points at day 8 post-infection, and then recovered by day 14. Administering low, medium, and high doses of FYQHD from day 4–10 post-infection helped alleviate the weight loss in PR8-infected mice. And OSV showed a protective effect on mice too (Figure 1A). Therefore, FYQHD demonstrated a significant protective effect on the body weight of PR8-infected mice, with the low dose proven to be effective (*P* < 0.0001). Subsequent experiments were conducted using the low dose of FYQHD (24 g/kg).

We then used mouse lung indices and H&E staining to evaluate the lung injury of flu mice. The mice of PR8 group showed significantly higher lung indices compared to those of PBS group. However, the administration of FYQHD or OSV notably reduced the lung indices caused by viral infection (Figure 1B). The H&E staining results showed normal lung tissue morphology in the lungs of PBS group. On day 7 post-infection, the lung tissues of PR8 group displayed collapsed alveolar structures, thickened alveolar walls, extensive infiltration of inflammatory cells, and exudates in the airway lumen. Treatment with FYQHD or OSV resulted in reduced infiltration of inflammatory cells and exudates in the airway lumen, as well as preserved alveolar structure (Figure 1C).

### 3.3 FYQHD protects against influenza a virus infection by reducing viral load and oxidative stress

**FIGURE 2:**
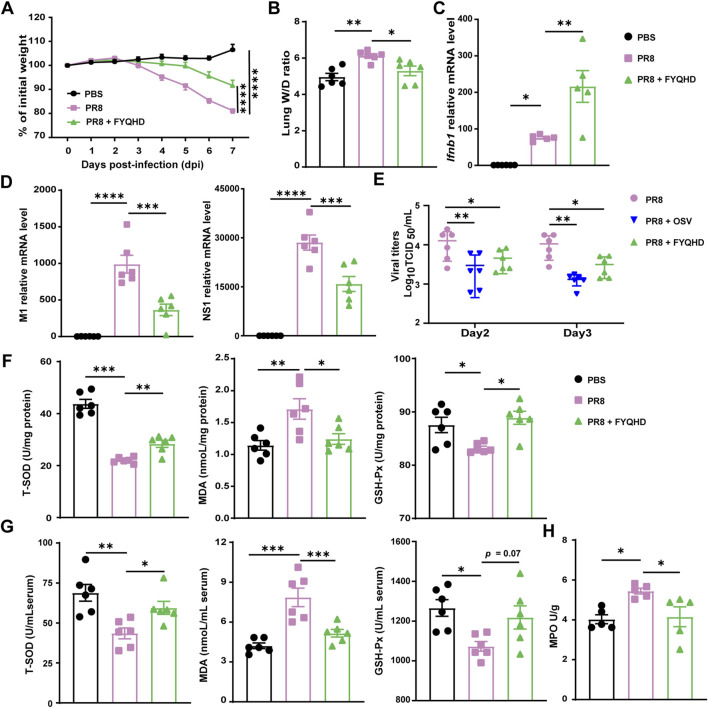
Oral FYQHD administration attenuated viral load and oxidative stress in the lungs upon IAV infection. **(A)** Percentage of weight loss post infection relative to initial body weight (day 0) (n = 6 - 7). **(B)** Lung W/D ratio of mice (n = 6). **(C)** The expression of *Ifnb1* detected in lung tissues by real-time PCR (n = 5). **(D)** Relative PR8 M1 and NS1 gene expression detected in lung tissues by real-time PCR (n = 6). **(E)** The virus titers in lung tissues of flu mice on 2 and 3 dpi were measured by TCID_50_ using MDCK cells (n = 6). **(F)** Concentrations of T-SOD, MDA, and GSH-Px in lungs from each group (n = 6). **(G)** Concentrations of T-SOD, MDA, GSH-Px in the sera from each group (n = 6). **(H)** Concentrations of MPO in the lungs from each group (n = 6). Data were presented as means ± SEM. Statistical significance was determined using one-way ANOVA, followed by Tukey test. *P < 0.05, **P < 0.01, ***P < 0.001, ****P < 0.0001 vs. PR8 group.

The results demonstrated that with the infection of influenza virus, the mice of PR8 group exhibited substantial weight loss compared to non-infective group. And the weight loss can be effectively reversed by FYQHD administration (Figure 2A). The lung wet/dry ratios can assess the edema of lung tissues visually. The mice of PR8 group showed considerably higher lung wet/dry ratios than those of PBS group. Meanwhile, FYQHD treatment reduced the lung wet/dry ratio of mice significantly (Figure 2B).

Within 1 week post-infection, the lung tissues of mice with influenza experienced extensive viral replication. Our results also showed that FYQHD treatment reduced IAV replication in lungs, as indicated by the reduced expression of viral non-structural protein 1 (NS1) and viral M1 gene (Figure 2D). Consistently, the examination of viral titers on day 2 and day 3 post-infection showed that both OSV and FYQHD treatment successfully reduced the viral loads of the lungs from IAV mice (Figure 2E). Importantly, FYQHD administration increased the expression of *Ifnb1*and *Ifna1* in lungs compared to those of PR8 group on 7 dpi (Figure 2C; Supplementary Figure S7A). Therefore, we proposed that FYQHD regulated IAV replication probably by enhancing IFN-I expression in lung tissues.

To assess the influence of FYQHD on oxidative stress, total superoxide dismutase (T-SOD), glutathione peroxidase (GSH-Px), and malondialdehyde (MDA) in the sera and lungs were measured. Oral administration of FYQHD significantly improved the concentrations of T-SOD and GSH-Px while reducing MDA levels in the sera and lungs of mice compared to those of PBS controls (Figures 2F, G). Similarly, PR8 drastically elevated the concentration of MPO in the lungs of mice, which was notably decreased by FYQHD treatment (Figure 2H). Hence, FYQHD showed strong antiviral and antioxidant effects during IAV infection.

### 3.4 FYQHD protected intestinal barrier against damage in flu mice

**FIGURE 3:**
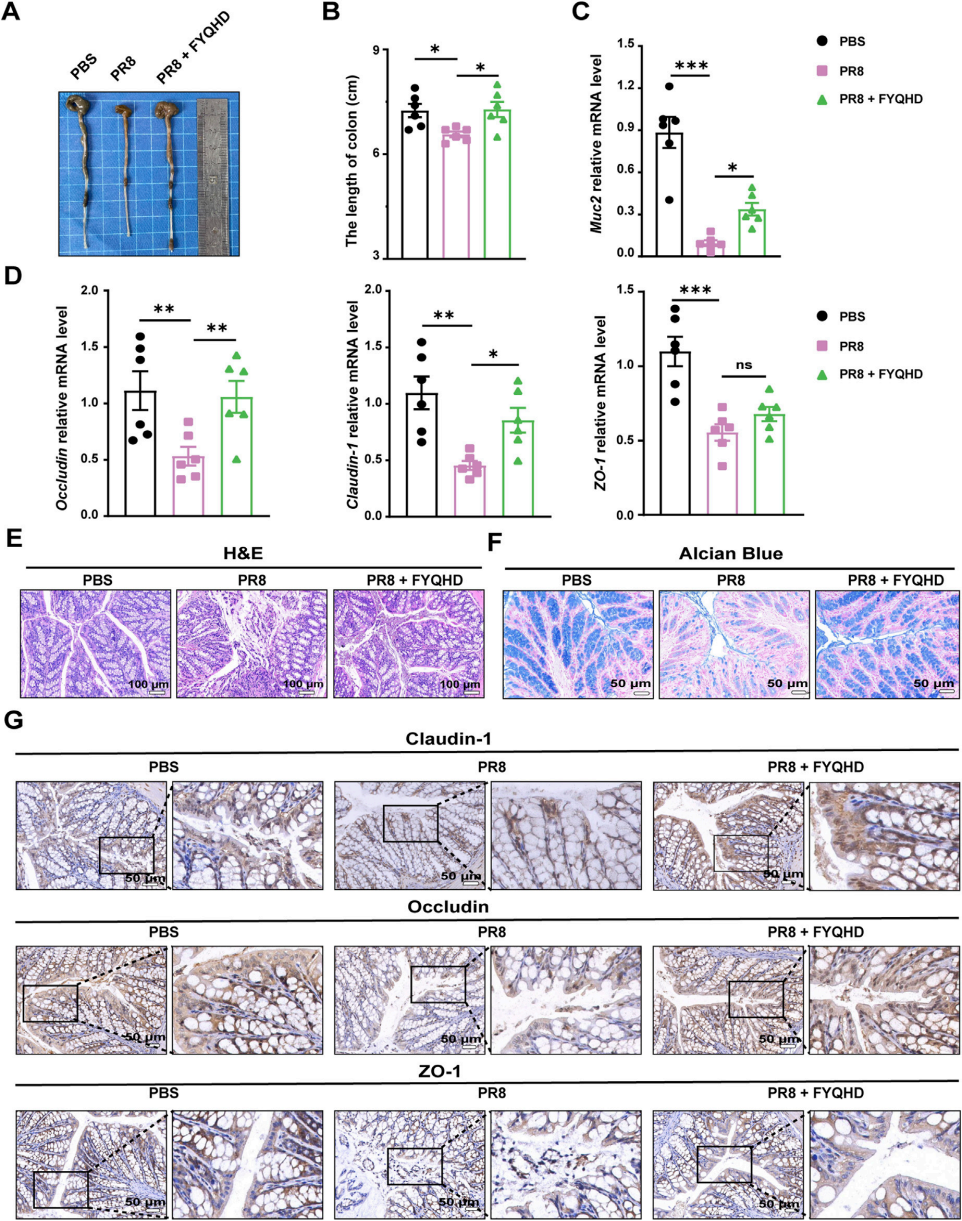
Protection of gut barrier damage in colons of flu mice by FYQHD. **(A, B)** Representative pictures of colons and colon lengths (n = 6). **(C, D)** Expressions of gut barrier-related molecules, *Muc2, Occludin, Claudin-1* and *ZO-1* in colon tissues detected by RT-qPCR. Data were presented as means ± SEM (n = 6 per group). Statistical significance was determined using one-way ANOVA, followed by Tukey test. *P < 0.05, **P < 0.01, ***P < 0.001, ****P < 0.0001 vs. PR8 group. **(E)** Representative microscopic pictures of H&E staining. (Scale bar: 100 μm). **(F)** Representative images of Alcian blue-stained inner mucus layer of colonic sections. (Scale bar: 50 μm). **(G)** Immunohistochemical staining of Occludin, Claudin-1 and ZO-1 in colons of different groups. (Scale bar: 50 μm).

The colon length of PR8-infected mice was significantly shorter than those of PBS group on 7 dpi. FYQHD treatment restored the length of the colons (Figures 3A, B). Based on this observation, we examined the influence of FYQHD on the integrity of the intestinal barrier of flu mice. H&E staining served as a crucial indicator to evaluate the extent of colonic mucosa injury. As depicted in Figure 3E, compared to those of PBS group, the integrity of the intestinal mucosal structure was destroyed clearly in PR8 group. This was evidenced by incomplete crypt structures and disorganized villi with noticeable fractures and localized necrosis ranging from mild to severe. In contrast, FYQHD treatment rectified these pathological changes. Goblet cells in the colonic epithelium that secrete mucin were assessed utilizing Alcian blue staining. PR8 infection led to a substantial reduction in the thickness of the colonic epithelial mucosa and the amount of intestinal glycoprotein mucins. Remarkably, the administration of FYQHD exhibited significant restoration of these pathological changes (Figure 3F).

As we know, there are important protein components of the intestinal barrier related to intestinal integrity, including the tight junction proteins Claudin-1, ZO-1 and Occludin. The reduced expression of these proteins results in the destruction of the intestinal mucosa, arousing inflammatory response. As detected by qPCR, the expression of *Muc2, Occludin, Claudin-1*, and *ZO-1* displayed a noteworthy decrease in the colon tissues of PR8-infected mice. However, FYQHD intervention notably reversed their expression (Figures 3C, D). Consistently, immunohistochemical staining revealed the corresponding decrease of Claudin-1, ZO-1, and Occludin in the colons of PR8-infected mice. Encouragingly, FYQHD treatment effectively prevented the deteriorating loss of protein expression, and almost restored their expression to normal levels. The tight junctions between the intestinal epithelial cells were effectively maintained (Figure 3G).

These results implied that FYQHD may preserve the integrity of the intestinal barrier by restoring the expression of tight junction proteins. This allows for the maintenance of intestinal goblet cells in mice, highlighting the protective capacity of FYQHD on the intestinal barrier.

### 3.5 FYQHD treatment regulated the composition of gut microbiota

**FIGURE 4:**
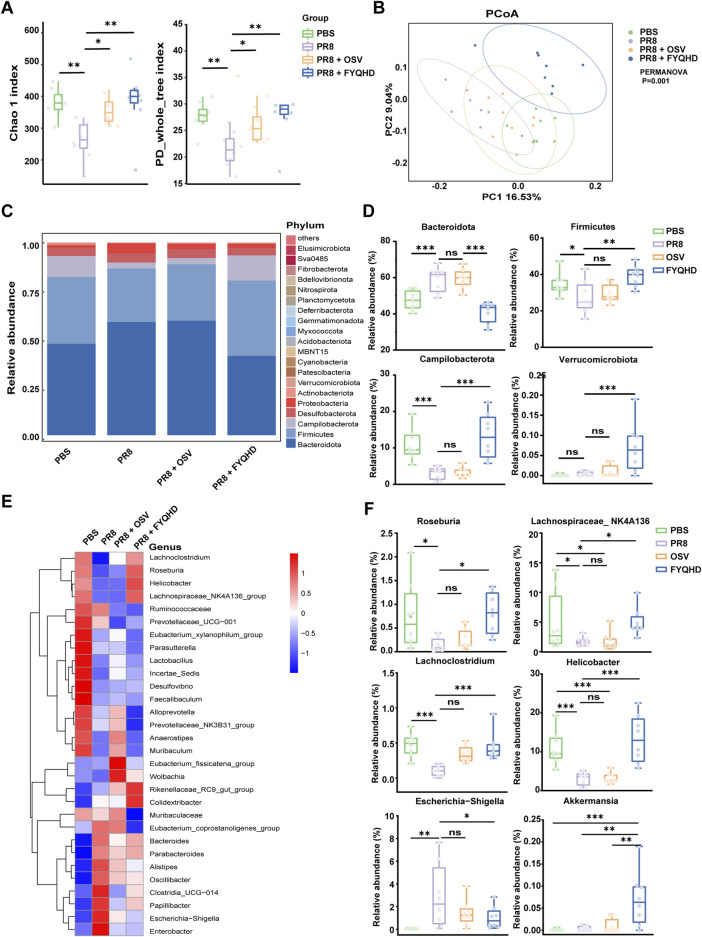
Influence of PR8 infection and FYQHD treatment on the homeostasis of intestinal microbiota in mice. **(A)** Alpha diversity upon oral therapy represented by the Chao1 and PD whole-tree indices. **(B)** Principal coordinate analysis (PCoA) plots upon different treatment assessed by PERMANOVA for β diversity. **(C)** Microbial community composition and the relative abundance for top 30 microbes at the phylum level. **(D)** Relative abundances of representative bacteria at phylum levels. **(E)** Relative abundance for top 30 microbes at the genus level. **(F)** Relative abundances of representative bacteria at genus levels. n = 8 for each group. Statistical analysis was performed using the one-way ANOVA. *P < 0.05, **P < 0.01, ***P < 0.001 vs. PR8 group.

The influence of FYQHD on the gut microbial composition of PR8-treated mice was further investigated using 16S rRNA sequencing. The α diversity measure was employed to assess the richness and diversity of the gut microbiota. Several indices, including Chao1 and PD whole-tree index (Figure 4A), demonstrated comparable trends, suggesting that both OSV and FYQHD treatment led to the maintenance of the diversity of gut microbiota, resulting in levels more closely resembling those of the PBS group. The dissimilarity of β-diversity was assessed using principal coordinate analysis (PCoA) (Figure 4B). PCoA using the unweighted UniFrac dissimilarity index revealed distinct clustering, PR8 group exhibited a noticeable separation from PBS group, whereas FYQHD and OSV treatment groups were demonstrated to be closer to PBS group (PERMANOVA *P* = 0.001). In general, the administration of FYQHD or OSV significantly changed the structural microbial diversity in the intestines of PR8-infected mice.

Subsequently, we evaluated the variances in populations and abundances among the 4 groups. The demonstrated top 30 microbes at the phylum (Figure 4C) and genus (Figure 4E) levels highlighted significant alterations within the composition of gut microbiota. At the phylum level (Figures 4C, D), *Bacteroidota*, *Firmicutes* and *Campilobacterota* were predominant phyla in the 4 groups. In PR8-infected mice, there was a notable increase in the relative abundance of *Bacteroidotas*, accompanied by a decrease in the levels of *Firmicutes* and *Campilobacterota*. However, the treatment of FYQHD effectively reversed these trends, restoring them to the levels comparable to those observed in PBS group. Furthermore, *Akkermansia* is well known to play a critical role in preserving intestinal mucosal function and is the sole enteric bacterium belonging to the *Verrucomicrobiota* phylum (Rajilic-Stojanovic and de Vos, 2014). As depicted in Figure 4D, there was a substantial increase in *Verrucomicrobiota* following FYQHD treatment, consistent with the initial findings of elevated intestinal glycoprotein mucins after FYQHD treatment as displayed in Figures 4C, E. At the genus level, there were remarkable changes in the levels of various gut microbiota in PR8-infected mice (Figure 4E). The infection of influenza virus decreased the relative abundance of the genera of *Roseburia*, *Lachnospiraceae_NK4A136*, *Lachnoclostridium*, and *Helicobacter*, while increased the level of genera of *Escherichia−Shigell*a. The treatment of FYQHD reinstated the composition of the gut microbiota to a level similar to that of the PBS group (Figure 4F). More notably, linear discriminant analysis effect size analysis (LEfSe) generated distribution histograms of LDA values, as illustrated in Supplementary Figure S3 (LDA >3, *p* < 0.05). Following FYQHD treatment, a number of bacterial genera such as *Helicobacter*, *Rikenellaceae_RC9*, *Roseburia*, and *Lachnospiraceae_NK4A136* exhibited enrichment, suggesting their potential as biomarkers for the FYQHD treatment group. Overall, FYQHD treatment significantly remodeled the composition and abundance of the gut microbiota and reversed the gut dysbiosis induced by PR8 infection.

### 3.6 FYQHD treatment altered microbiota-derived short-chain fatty acid (SCFA) profiles

**FIGURE 5:**
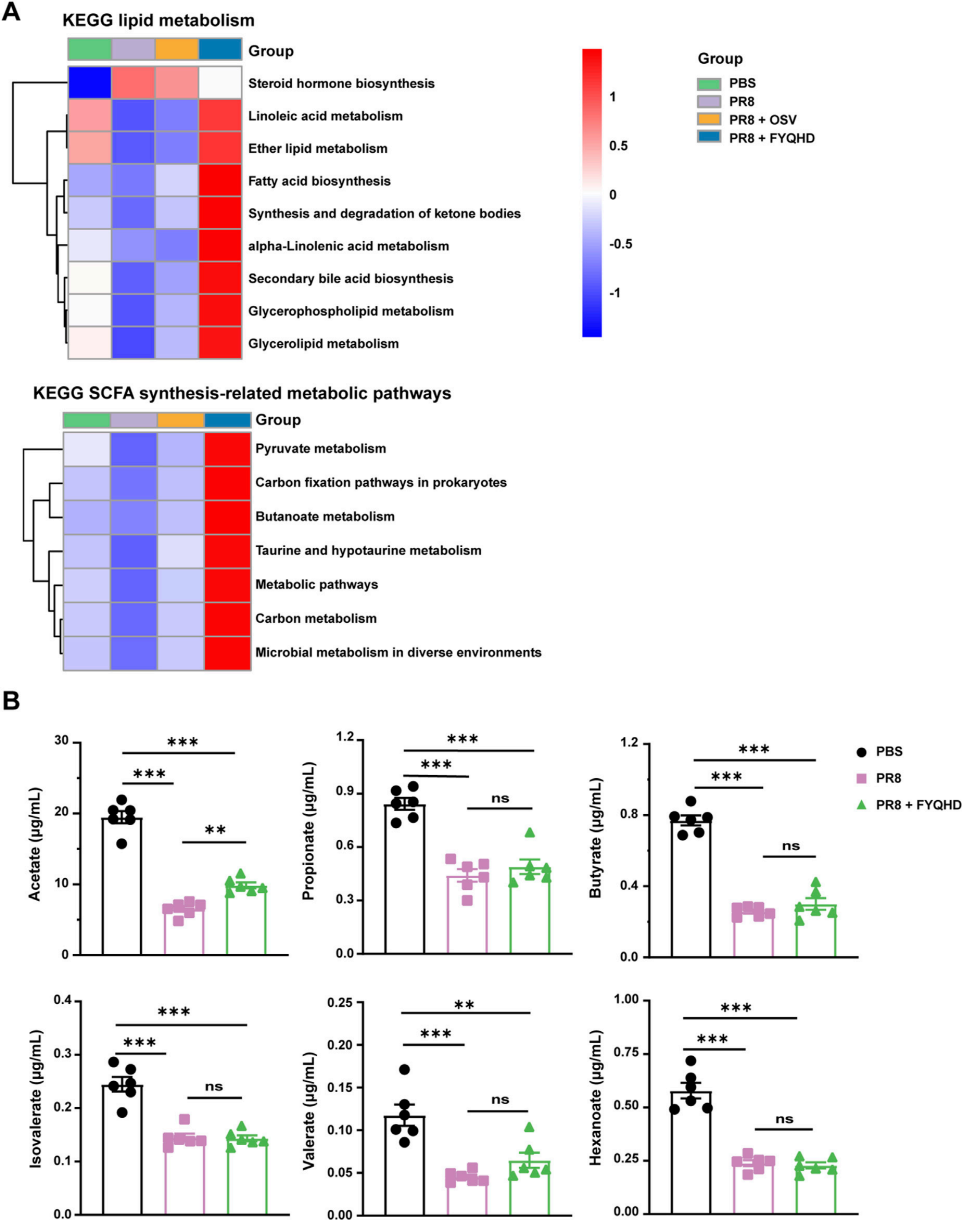
FYQHD regulated SCFA production of the gut microbiota in flu mice. **(A)** Analysis of differences in the Kyoto Encyclopedia of Genes and Genomes metabolic pathway. **(B)** Serum SCFA concentrations from mice of PBS, PR8 and PR8 + FYQHD groups (n = 6). Data were presented as means ± SD. *P* values were calculated by ANOVA followed by Tukey’s test, *P < 0.05, **P < 0.01, ***P < 0.001, ns indicates *P > 0.05* vs. PR8 group.

Previous 16S rRNA sequencing analysis showed that gut microbiota in FYQHD-treated group displayed a predominance of the genera *Akkermansia, Lachnoclostridium, Roseburia*, and *Lachnospiraceae_NK4A136* group, which were generally associated with SCFA metabolism (Kasahara et al., 2018; Ma et al., 2020; Yang et al., 2021; Cani et al., 2022). SCFA represent the final products of anaerobic fermentation within the gastrointestinal tract and play a critical role in preserving immune homeostasis within the intestine (Lavelle and Sokol, 2020). The enrichment analysis of KEGG pathways was predicted from the results of 16S rRNA sequencing. Analysis of the KEGG metabolic pathways demonstrated that FYQHD predominantly enhanced lipid metabolic pathways, with the exception of steroid hormone biosynthesis. Furthermore, the analysis of KEGG metabolic pathways revealed that FYQHD notably enhanced metabolic pathways associated with the synthesis of SCFAs, such as pyruvate, carbon fixation pathways in prokaryotes, butanoate metabolism, taurine and hypotaurine metabolism, and carbon metabolism (Figure 5A). To further investigate the impact of FYQHD on the peripheral circulation of SCFAs, the amounts of acetate, propionate, butyrate, valerate, isobutyrate, and isovalerate in sera were measured. Interestingly, the acetate levels were found to be significantly elevated in sera from FYQHD-treated mice compared to PR8-infected mice, whereas concentrations of the other 5 SCFAs did not exhibit statistically significant differences (Figure 5B). Collectively, the administration of FYQHD resulted in changes in gut microbiota and increased synthesis of SCFAs in mice with influenza. These results indicated that acetate, a metabolite derived from gut microbiota, may play a crucial role in the therapeutic mechanism of FYQHD.

### 3.7 The protective effect of FYQHD on lung injury was abolished by antibiotic pretreatment

**FIGURE 6:**
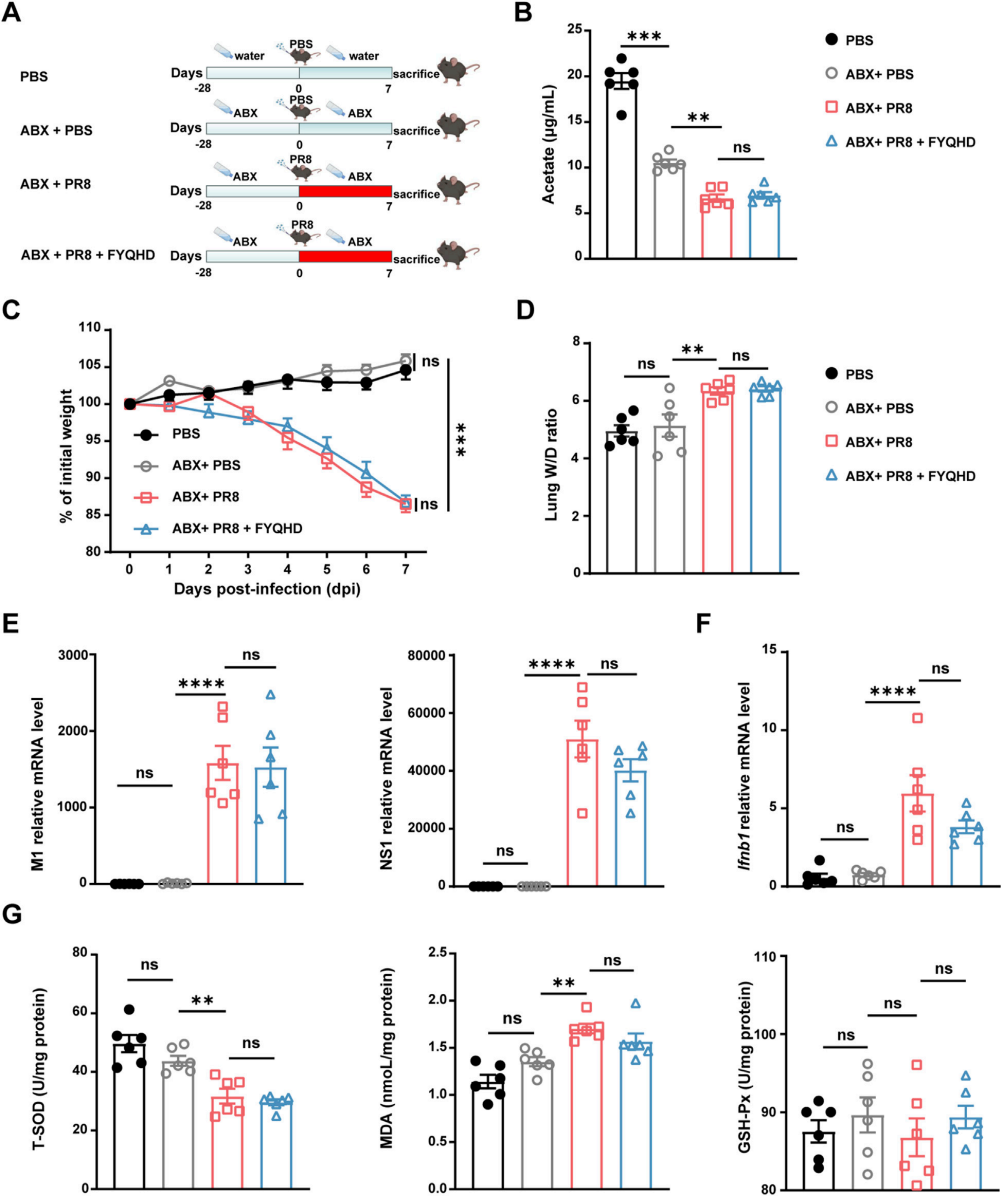
The protective effect of FYQHD on the lungs upon IAV infection was abrogated by antibiotic pretreatment. **(A)** Experimental schematic diagram. **(B)** Serum acetate concentrations from mice of PBS, ABX + PBS, ABX + PR8 and ABX + PR8+ FYQHD groups (n = 6). **(C)** Percentage of weight loss post infection relative to initial body weight (day 0) (n = 6 - 7). **(D)** Lung W/D ratio of mice (n = 6). **(E)** The relative gene expression of M1 and NS1 of PR8 detected in lung tissues by real-time PCR (n = 6). **(F)** *Ifnb1* gene expression detected in lung tissues by real-time PCR (n = 6). **(G)** Concentrations of T-SOD, MDA and GSH-Px in lungs from each group (n = 6). Data were presented as means ± SEM. Statistical significance was determined using one-way ANOVA, followed by Tukey test. **P < 0.01, ****P < 0.0001, ns indicates *P > 0.05* vs. ABX + PR8 group.

To investigate whether gut microbiota and the acetate participated in the protective effect of FYQHD against influenza-induced pulmonary damage, wild-type mice were given an antibiotic cocktail (ABX) in drinking water 28 days before PR8 inoculation (Figure 6A). After 4 weeks of antibiotic treatment, it was observed that FYQHD treatment did not increase acetate levels in the sera of mice (Figure 6B). Similarly, FYQHD did not change the levels of the remaining 5 SCFAs (Supplementary Figure S4). Strikingly, the ABX + PR8 + FYQHD group of mice exhibited similar severity of lung injury to those in ABX + PR8 group. This was supported by the indistinguishable body weight change (Figure 6C) and lung wet/dry ratios (Figure 6D). Furthermore, antiviral responses in ABX + PR8 + FYQHD group exhibited comparable trends to those in ABX + PR8 group, as evidenced by similar levels of lung viral load (Figure 6E) and *Ifnb1 and Ifna1* expression (Figure 6F; Supplementary Figure S7B). Meanwhile, the impact of FYQHD treatment on oxidative stress responses of gut microbiota-depleted mice was further investigated. The concentration of T-SOD, GSH-Px, and MDA in the lungs (Figure 6G) and sera (Supplementary Figure S5) displayed no significant difference between ABX + PR8 + FYQHD and ABX + PR8 groups. From the results mentioned above, it can be concluded that the presence of gut microbiota was indispensable for the protective effects of FYQHD on the lung injuries in PR8-infected mice.

### 3.8 FYQHD did not ameliorate the intestinal damage in flu mice with depleted gut microbiota

**FIGURE 7:**
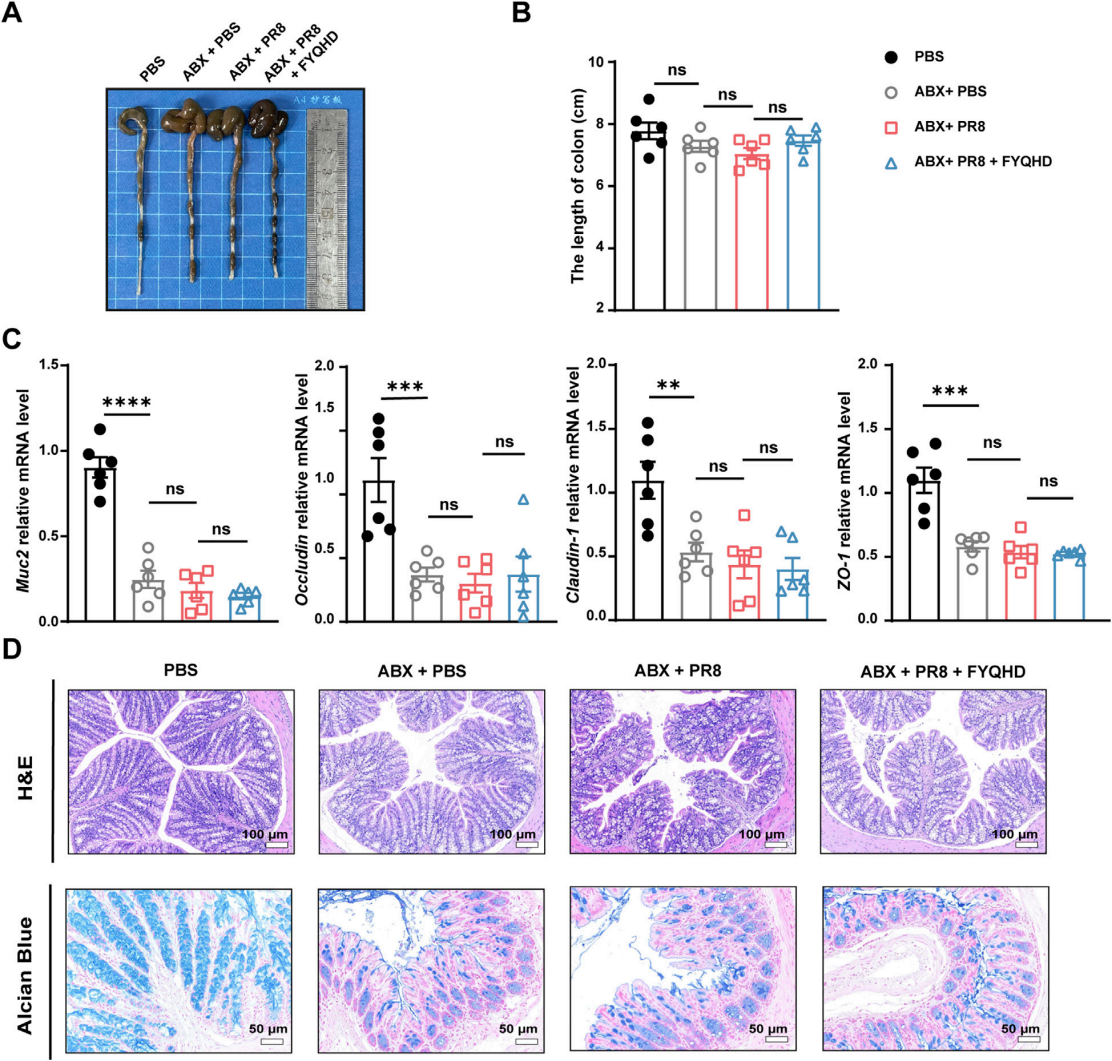
Protection of gut barrier damage in the colons of flu mice by FYQHD was abolished by antibiotic pretreatment. **(A, B)** Representative pictures of colons and colon lengths (n = 6). **(C)** The mRNA expressions of gut barrier-related molecules, *Muc2, Occludin, Claudin-1*, and *ZO-1* in colon tissues were detected by RT-qPCR. Data were presented as means ± SEM (n = 6 per group). Statistical significance was determined using one-way ANOVA, followed by Tukey test. **P < 0.01, ***P < 0.001, ****P < 0.0001, ns indicates *P > 0.05* vs. ABX + PR8 group. **(D)** Representative microscopic pictures of H&E staining (Scale bar: 100 μm) and representative images of Alcian blue-stained inner mucus layer of colonic sections. (Scale bar: 50 μm).

Whether microbiota and acetate were involved in the protection of FYQHD against intestinal damage in flu mice were investigated. As shown in Figures 7A, B, the 3 ABX-pretreated groups exhibited pronounced cecal oedema compared to those of PBS group, while no significant difference in colon lengths was observed. Strikingly, ABX + PR8 + FYQHD group mice displayed similar severity of intestinal barrier damage to ABX + PR8 group, as evidenced by indistinguishable expression of gut integrity markers *Muc2, Occludin, Claudin-1*, and *ZO-1* (Figure 7C). Meanwhile, the therapeutic effect of FYQHD was not observed in flu mice under the circumstances of gut microbiota depletion. Specifically, results of H&E staining, Alcian blue staining (Figure 7D), and immunohistochemical staining (Occludin, Claudin-1, and ZO-1) (Supplementary Figure S6) determinations revealed no obvious differences between the mice of ABX + PR8 + FYQHD group and ABX + PR8 group, suggesting that the absence of gut microbiota counteracted the beneficial effects of FYQHD. These experiments strongly suggested that the protection provided by FYQHD against intestinal barrier damage caused by PR8 infection relies on the homeostasis of gut microbiota.

### 3.9 Acetate suppresses IAV replication by enhancing IFN-I production in lung epithelial cells

**FIGURE 8:**
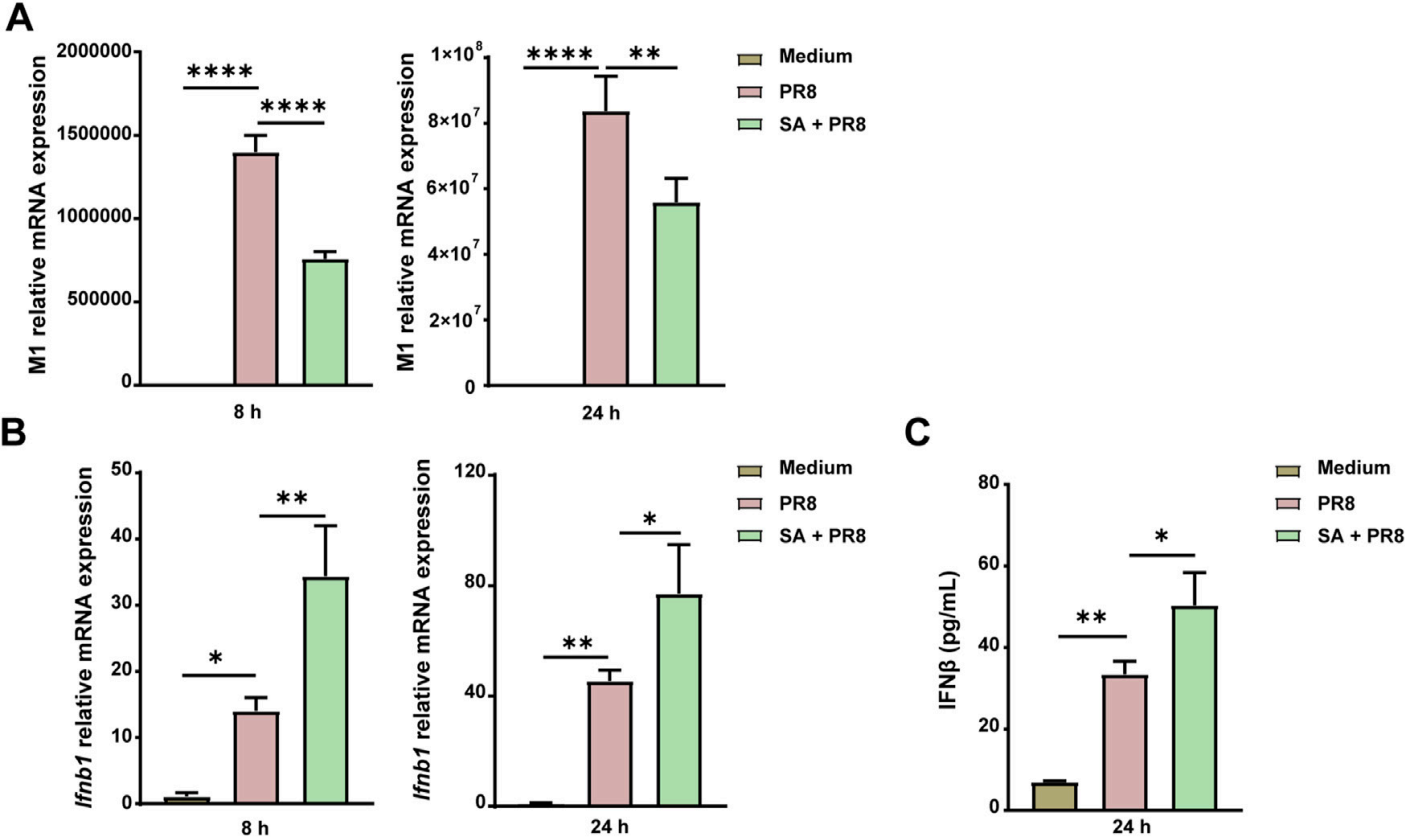
Acetate controls IAV replication by enhancing the expression of IFN-I in A549 cells. A549 cells were pretreated with 260 µM SA for 24 h and then infected with PR8 (MOI = 2) for 8 h or 24 h. **(A)** Relative PR8 M1 gene expression in A549. **(B)** Relative *Ifnb1* gene expression in A549. **(C)** Concentrations of IFN-β in the cell supernatant 24 h after infection were detected by ELISA. Data represented mean ± SD of 3 independent experiments. * *P* < 0.05, **P < 0.01, ****P < 0.0001.

Given the notable decline in viral load observed in the lungs and increased levels of acetate in the sera of PR8 + FYQHD group compared to PR8 mice, we hypothesized that acetate may exert a direct antiviral effect on pulmonary cells. Human lung epithelial cells (A549) were exposed to 260 µM sodium acetate (SA) 24 h before the infection of PR8. A549 cells treated with acetate showed a significant reduction in IAV replication at 8 h and 24 h post-infection, as evidenced by decreased expression of the viral M1 gene (Figure 8A). Remarkably, acetate treatment enhanced the expression of *Ifnb1* in A549 cells at 8 and 24 h post-IAV infection (Figure 8B) and increased IFN-β levels at 24 h post-IAV infection (Figure 8C). These findings suggest that the antiviral protective effect of FYQHD against IAV infection may be partially mediated by increased acetate levels and subsequent IFN-β production.

## 4 Discussion

FYQHD is a traditional Chinese medicine formula used clinically to treat influenza by experienced clinicians at Shuguang Hospital, Shanghai University of Traditional Chinese Medicine. In the current study, the therapeutic effects of FYQHD on influenza were thoroughly demonstrated, including the inhibition of viral replication, alleviation of oxidative stress, amelioration of lung injury, and protection of survival in PR8-infected mice. The mechanisms are likely associated with restoring the balance of gut dysbiosis, increasing the enrichment of SCFA-producing bacteria, and upregulating the production of gut microbiota-derived acetate, which protected mice against IAV infection by enhancing IFN-I expression.

Our study showed that FYQHD improved the survival of mice with influenza. Mice in PBS group appeared lively with shiny fur, normal activity levels, consistent food and water intake, and gained body weight steadily. From 3 dpi on, the infected mice in PR8 group began to show flu symptoms, like messy and dull fur, decreased activity, lower food and water intake, experienced convulsions, hunchbacked, piloerection and shortness of breath, etc. In mice, infection with influenza virus resulted in body weight loss. The timely administration of FYQHD to treat flu has been demonstrated notably effective in attenuating viral replication and achieving therapeutic goals, including the recovery of body weights and decreased lung dry/wet weight ratios (D/W). Evidence has proven that ROS significantly increases in lung epithelial cell lines and primary murine cells upon IAV infection (Amatore et al., 2015). The nonstructural protein 1 (NS1) plays a significant role in the stimulation of ROS production by modulating the dynamics of mitochondria (Lee et al., 2021). Elevated levels of ROS facilitate IAV replication, thereby enhancing its pathogenesis (Peterhans, 1997). The influenza virus triggers an overproduction of ROS, ultimately exacerbating lung damage. FYQHD administration promoted the production of SOD and GSH-Px in mice with influenza while simultaneously reducing MDA levels, potentially alleviating the adverse effects of oxidative stress on lung injury.

FYQHD exhibited protective efficacy against influenza-associated intestinal damage in mice. While the H1N1 virus does not directly infect or replicate in human intestinal tissues, cytokines released by immune cells (e.g., Th17 cells) can lead to intestinal damage following IAV infection (Wang et al., 2014). Upon influenza virus infection, respiratory mucosa-derived lymphocytes migrate to the mesenteric lymph nodes via a chemokine-receptor (CCL25-CCR9) axis, disrupting the homeostasis of intestinal microbiome and ultimately causing intestinal immune damage (Ahmadi Badi et al., 2021). In our study, the colon lengths of PR8-infected mice were found to be much shorter than those of normal mice. However, FYQHD partially restored colon lengths to levels comparable to those of the PBS group. Consistent with our results, Wang *et al.* found that respiratory influenza virus infection resulted in intestinal immune damage and a decrease in colon length (Wang et al., 2014). Furthermore, other studies have shown that flu-infected mice experienced damage to both the lungs and intestines (Zhu et al., 2018). Our experiment indicated that the influenza virus significantly reduced the expression of the tight junction proteins Claudin-1, ZO-1, and Occludin, directly decreasing the integrity and causing damage to the intestinal epithelial barrier. Concurrently, the expression of *Muc2* was significantly reduced by influenza virus. Muc2 mucin is crucial for protecting the gut barrier and maintaining the balance of gut microbiota (Liu et al., 2020). Interestingly, FYQHD treatment ameliorated the aforementioned gut injury. Further research is needed to investigate the mechanism of FYQHD on PR8-induced intestinal injury.

Furthermore, evidence indicated that the gastrointestinal tract and respiratory system, along with their respective microbiomes, were interconnected and mutually influenced. This concept is known as the gut-lung axis (Dumas et al., 2018). The ancient theory in traditional Chinese medicine that describes the “the lung and the large intestine are interior-exterior” is consistent with the modern concept of the “gut-lung axis” as revealed by contemporary research. Thus, the gut microbiota has become a new therapeutic target for respiratory viral infection currently. Certain herbal medicines were reported to modulate the diversity and abundance of gut microbiota, which could have played a crucial role in inhibiting IAV replication and reducing inflammatory damage to lung tissues, such as GeGen QinLian decoction (Deng et al., 2021), Qingfeiyin decoction (Li et al., 2022), and Cangma Huadu granules (Liu et al., 2022). Hence, we proceeded to examine whether FYQHD regulated the composition of gut microbiota during IAV infection. The results demonstrated that IAV infection had a significant impact on the balance of the gut microbiota, as indicated by a decrease in the diversity and richness of the microbiota. In addition, IAV infection significantly increased the abundance of *Escherichia–Shigella*, while reduced the abundance of *Lachnospiraceae_NK4A136*, *Lachnoclostridium, Roseburia*, and *Helicobacter* in the mouse gut microbiota. Nevertheless, FYQHD treatment reversed these changes. Earlier studies had suggested that *Escherichia–Shigella*, a bacterium with conditional pathogenicity in the intestine, was a common trigger for vomiting and diarrhea (Wang et al., 2014). *Helicobacter* is thought to provide effective protection for mice against tissue inflammatory damage in asthma, easing the infiltration of inflammatory cells in the bronchioles and alveoli (Arnold et al., 2011). It is well-known that SCFAs are crucial for maintaining the homeostasis of the intestinal epithelial barrier (Parada Venegas et al., 2019). As demonstrated here, the abundance of *Lachnoclostridium, Roseburia*, and *Lachnospiraceae_NK4A136* were notably increased by FYQHD treatment, and they were all considered beneficial members *via* regulating SCFA metabolism (Kasahara et al., 2018; Ma et al., 2020; Yang et al., 2021). The abundance of *Lachnospiraceae* was significantly decreased in the intestines of COVID-19 patients, replaced by other potentially pathogenic bacteria, contributing to pulmonary hyperinflammation (Kaushal and Noor, 2022). In a recent study, mice that received *Lachnospiraceae* showed reduced airway RSV viral load, diminished lung injury, and increased overall lifespan (Antunes et al., 2019). *Akkermansia*, a well-established next-generation probiotic and producer of SCFAs, has been demonstrated to prominently feature in the connections between gut microbes and host responses (Bae et al., 2022). It contributed to an anti-H7N9 effect through its ability to regulate inflammation and the immune system (Hu X. et al., 2020). Interestingly, compared with the mice of PR8 group, the intestinal enrichment of *Akkermansia* was substantially promoted by FYQHD treatment in flu mice. Moreover, an important observation was that the modified microbiota diversity and composition after FYQHD administration appeared to align with those of PBS group. On the contrary, the change of gut microbiota in OSV-treated flu mice was comparable to those of PR8-infected mice. This was mainly because the primary anti-viral mechanism of OSV was the inhibition of neuraminidase, so it did not have a critical impact on the gut microbiota. These results implied that the employment of FYQHD could be an effective treatment of influenza by restoring the unbalanced gut microbiota, enhancing microbial diversity, and promoting the proliferation of beneficial bacteria.

Previous research had indicated that SCFA produced in the gut could enter the systemic circulation and exert distant biological effects (Sencio et al., 2020). Evidence showed that TCM had the potential to improve lipid metabolism by increasing SCFA levels and modulating the biosynthesis of branched-chain amino acids (Li et al., 2021). Our results indicated that SCFA-producing bacteria in the gut may play a crucial role in the protective effect of FYQHD during IAV infection. To determine if FYQHD exerts protective effects on lung injuries by promoting the production of SCFAs in the gut, the concentration of SCFAs in sera was measured after FYQHD treatment. As expected, FYQHD-treated group exhibited increased levels of acetate in the sera compared to those of PR8 group. Our findings were in an agreement with previous reports (Hashemi et al., 2017), showing that an increased abundance of the *Lachnospiraceae* family was associated with elevated serum levels of acetate. To further substantiate the hypothesis that the gut-lung axis was a target of FYQHD, this study generated mice with depleted gut microbiota using a broad-spectrum antibiotic. The results demonstrated that the protective effects of FYQHD on IAV infection were significantly diminished following gut microbiota depletion. Measures of acetate, viral loads, *Ifnb1*, and oxidative stress factors were similar in both ABX + PR8 group and the ABX + PR8 + FYQHD group. Additionally, no significant differences were observed in intestinal histological injuries and the expression of tight junctions between these groups. We fully acknowledge that traditional Chinese medicine might operate through multiple targets via various ingredients entering the bloodstream or their metabolic products. Germ-free mouse models and additional experiments are necessary to further validate these results. It is possible that other protective effects, such as anti-inflammatory actions, may not be adequately demonstrated in this model.

Emerging evidence suggested that acetate, a microbiota-derived metabolite, influenced immune responses during viral infection in the lung. Recently, research demonstrated that acetate protected against RSV infection by boosting type 1 interferon responses in lung epithelial cells via GPR43 induction. Acetate administered orally enhanced IFN-β response by upregulating interferon-stimulated genes (ISGs) in the lung. The antiviral activity of acetate in pulmonary epithelial cell lines and its protective effect in RSV-infected mice relied on type 1 IFN signaling through the IFN-1 receptor (IFNAR) (Hashemi et al., 2017). It is well known that epithelial cells in the respiratory epithelium are the primary target cells for IAV replication (Ramos et al., 2019). Notably, our study showed that acetate treatment in A549 cells led to a significant enhancement of *Ifnb1* expression and a reduction in the replication of influenza A virus. Innate immune cells like macrophages and neutrophils play crucial roles in detecting IAV infection and suppressing the virus shortly after infection (Iwasaki and Pillai, 2014). A recent study highlighted the significance of acetate in macrophages in controlling IAV, as demonstrated by the decreased effectiveness of acetate after macrophage depletion (Niu et al., 2023). The study found that acetate from *Bifidobacterium pseudolongum NjM1* provided protection against influenza A virus infection by activating the GPR43-MAVS-IRF3 pathway and inducing the IFN-I signal. In summary, the data indicated a mechanism by which FYQHD boosted the presence of acetate-producing bacteria, resulting in heightened acetate concentrations in lungs. This facilitated the inhibition of IAV replication by augmenting IFN-I generation. Given the wide-ranging antiviral properties of IFN-I, FYQHD might also be effective against other viral infections, for example, SARS-CoV-2 or RSV.

There has been extensive research into TCM for the treatment of influenza, both as individual components and as TCM compound formulas. This study initially revealed the identification of 17 major compounds in sera following oral administration of FYQHD, including ephedrine, liquitin, baicalin, wogonoside, saikosaponin D, glycyrrhizic acid, wogonin, rhein, emodin, and so on. Increasing evidence has shown that baicalin can inhibit the replication of IAV *in vivo* and *in vitro*, as well as inhibit the inflammatory response (Ding et al., 2014; Jin et al., 2018; Pang et al., 2018). A prior investigation found that phillyrin could improve the survival of influenza mice, decrease the pulmonary viral titers and decreases lung indices (Qu et al., 2016). Glycyrrhizic acid is also demonstrated to enhance the survival rate of H1N1-infected mice, decreased the virus titers in lungs, and reduced the expression of inflammatory factors (Chen et al., 2015). Collectively, these studies indicated that multiple components of FYQHD might play a role in its anti-viral effects and protect against influenza through complicated mechanisms. However, further studies are still needed to verify which component of FYQHD is able to modulate the increase in the abundance of acetate-producing bacteria and consequently exhibiting an anti-viral effect.

FYQHD has demonstrated efficacy against community-acquired pneumonia (CAP) in clinical practice for nearly 2 decades. In our previous study, FYQHD showed efficacy in treating LPS challenge or carbapenem-resistant *Klebsiella pneumoniae* (CRKP) infection (Zhang et al., 2024). It displayed dual regulatory effects, dampening the inflammatory response by inhibiting the PI3K/AKT/mTOR/4E-BP1 and HMGB1/RAGE signaling pathways, while also boosting bacterial phagocytosis to manage bacterial burden in the liver and spleen. This research is the first to examine how the gut microbiome mediates the protective effects of FYQHD against influenza. However, this study has limitations, and additional research is necessary to determine how FYQHD specifically regulates microbiota function and intestinal barrier integrity. Our current findings demonstrate that FYQHD influences the composition and abundance of gut microbiota, enhancing populations of acetate-producing bacteria. Additional *in vivo* and *in vitro* studies are necessary to identify the precise compounds in FYQHD responsible for this biological activity. Our future work will focus on identifying the key active components of FYQHD and elucidating their roles in modulating gut microbiota and their metabolites. Another limitation is the lack of clinical data in this study. Fortunately, a randomized controlled trial (RCT) investigating the efficacy of FYQHD in influenza patients is currently underway. In future research, fecal and blood samples from influenza patients will be analyzed between FYQHD-treated and control groups to elucidate its protective role and underlying mechanisms.

## 5 Conclusion

**FIGURE 9:**
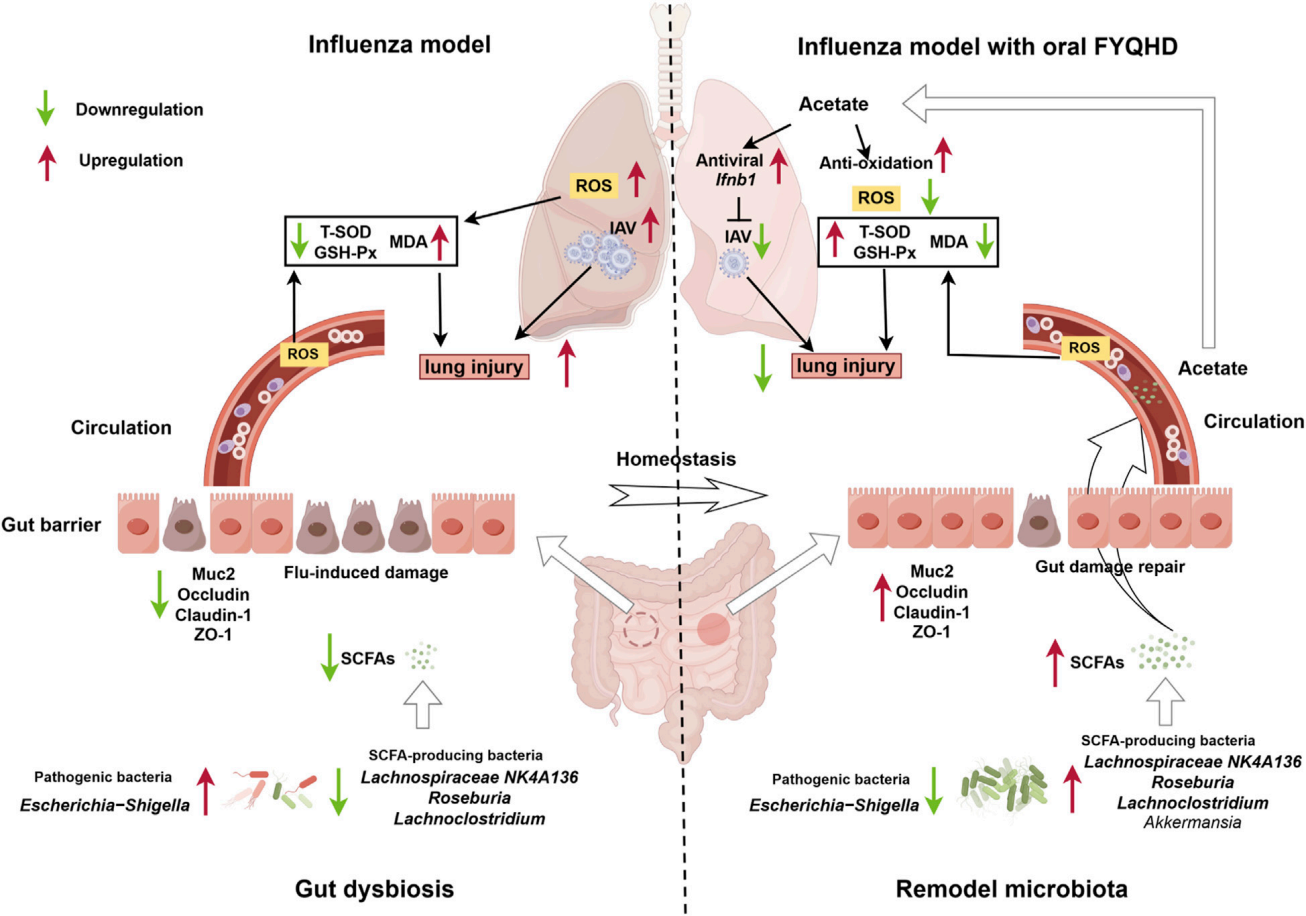
The administration of FYQHD remodeled the composition and diversity of gut microbiota, particularly up-regulated the abundance of SCFAs-producing bacteria such as *Lachnospiraceae_NK4A136, Lachnoclostridium, Roseburia and Akkermansia*. This led to increased production of acetate, which in turn promoted augmented IFN-I generation and antioxidative effects, resulting in the amelioration of lung injury (By Figdraw).

In conclusion, the present study demonstrated that the administration of FYQHD remodeled gut dysbiosis, particularly by increasing the abundance of acetate-producing bacteria such as *Lachnospiraceae_NK4A136*, *Lachnoclostridium*, and *Roseburia*, leading to higher acetate production. The acetate was transported to the lungs, where it stimulated IFN-β production, offering protection against IAV infection and enhancing the antioxidant stress response (Figure 9). These findings highlight the complex and critical relationship between the gut and lungs, providing an experimental basis for validating the concept of TCM based on the theory of ‘simultaneous treatment of the lungs and intestines.’ Our findings further support the clinical application of FYQHD and provide new insights into the mechanism of TCM for treating influenza.

## Data Availability

The raw 16S sequencing data is deposited at the NCBI repository, BioProject accession number PRJNA1120572. Available at: https://www.ncbi.nlm.nih.gov/bioproject/PRJNA1120572/.
