# Supplementary material for: Fei-Yan-Qing-Hua decoction attenuates influenza virus infection by enhancing host antiviral response through microbiota-derived acetate
Source: Front Pharmacol. 2024 Oct 10;15:1446749. doi: 10.3389/fphar.2024.1446749 (PMC11499185; doi:10.3389/fphar.2024.1446749)
Supplement: Supplementary file 1 [file DataSheet1.PDF]

Supplementary Material

Supplementary Table S1. Detailed information on nine traditional Chinese herbal medicines in the FYQHD.

Chinese name	English name	Scientific names	Amount (g)	Plant part	Lot number
Mahuang (麻黄)	Ephedra	*Ephedra sinica* Stapf	6	Stem	20230103-1
Shengdahuang (生大黄)	Rhubarb	*Rheum officinale* Bail.	6	Root and Rhizome	20221101-1
Jinqiaomai (金荞麦)	Buckwheat	*Fagopyrum esculentum* Moench	30	Rhizome	20221214-1
Gancao (甘草)	Licorice	*Glycyrrhiza uralensis* Fisch	9	Rhizome	220711
Huangqin (黄芩)	Radix scutellariae	*Scutellaria baicalensis* Georgi	9	Root	2022101308
Kuxingren (苦杏仁)	Semen armeniacae amarum	*Prunus armeniaca L.var.ansu* Maxim.	9	Seed	2022120307
Sangbaipi (桑白皮)	White mulberry	*Morus alba* L.	9	Root bark	20221105
Chaihu (柴胡)	Radix bupleuri	*Bupleurum chinense* DC.	9	Root	221114
Shengshigao (生石膏)	Gypsum	Gypsum	30	-	221229

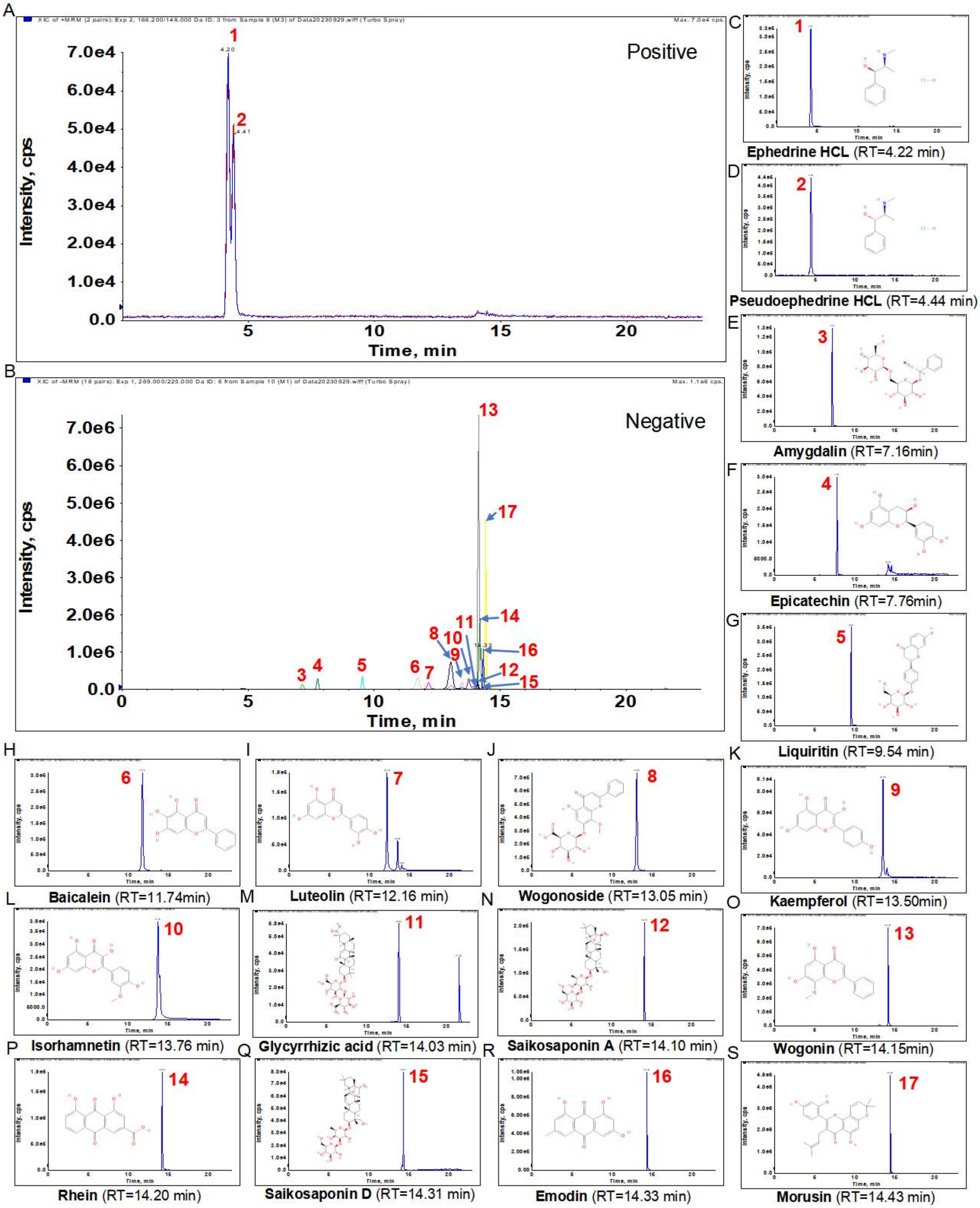

Supplementary Figure 1. Quantitative analysis of FYQHD and its representative compounds by UPLC-MS/MS. (**A**) TIC of positive ion model of FYQHD. (**B**) TIC of negative ion model of FYQHD. (**C-S**) Ephedrine (**C**), Pseudoephedrine (**D**), Amygdalin (**E**), Epicatechin (**F**), Liquiritin (**G**),

Baicalin (**H**), Luteolin (**I**), Wogonoside (**J**), Kaempferol (**K**), Isorhamnetin (**L**), Glycyrrhizic acid (**M**), Saikosaponin A (**N**), Wogonin (**O**), Rhein (**P**), Saikosaponin D (**Q**), Emodin (**R**) and Morusin (**S**).

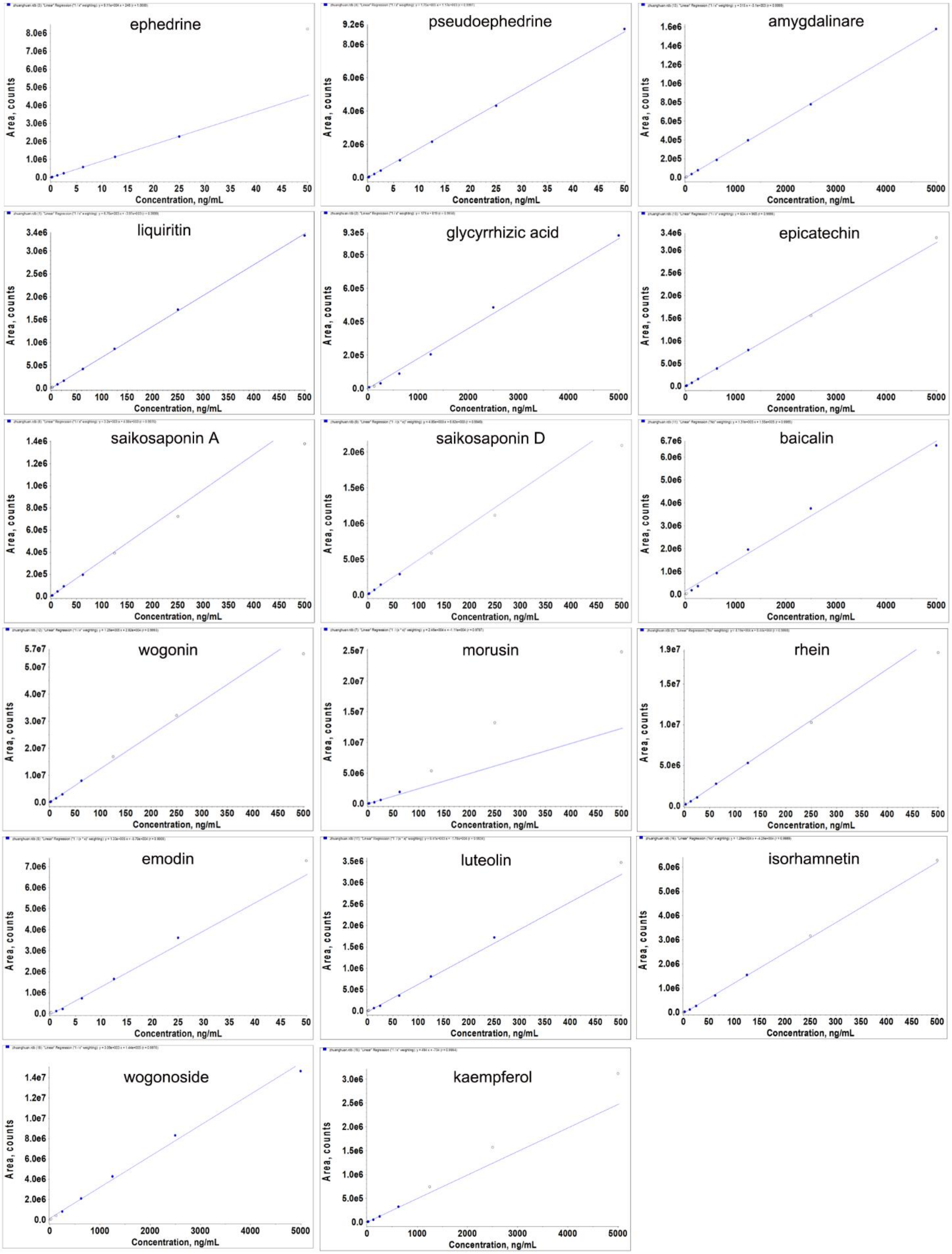

Supplementary Figure 2. The standard curve of standards used in UPLC-MS/MS.

Supplementary Table S2. Chromatograms data of compounds in FYQHD detected by HPLC-MS/MS.

Sign No.	t_R/min	Standards	Representative Herbs	Concentration of compound in FYQHD (ng/mg)	PubChem CID
1	4.22	Ephedrine	*Ephedra dahurica* Turcz. (Ephedraceae)	135	65326
2	4.44	Pseudoephedrine	*Ephedra dahurica* Turcz. (Ephedraceae)	113	9581
3	7.16	Amygdalin	*Prunus mandshurica* (Maxim.) Koehne. (Rosaceae)	1670	34751
4	7.76	Epicatechin	*Fagopyrum acutatum* Mansf. ex K.Hammer (Polygonaceae)	24.6	72276
5	9.54	Liquiritin	*Glycyrrhiza uralensis* Fisch. ex DC. (Fabaceae)	115	503737
6	11.74	Baicalin	*Scutellaria baicalensis* Georgi (Lamiaceae)	5530	5281605
7	12.16	Luteolin	*Scutellaria baicalensis* Georgi (Lamiaceae)	0.395	5280445
8	13.05	Wogonoside	*Scutellaria baicalensis* Georgi (Lamiaceae)	935	3084961
9	13.50	Kaempferol	*Morus alba var. tatarica* (L.) Loudon (Moraceae)	0.477	5280863
10	13.76	Isorhamnetin	*Fagopyrum acutatum* Mansf. ex K.Hammer	0.411	5281654

			(Polygonaceae)		
11	14.03	Glycyrrhizic acid	*Glycyrrhiza uralensis* Fisch. ex DC. (Fabaceae)	81.6	14982
12	14.10	Saikosaponin A	*Bupleurum chinensis* DC. (Apiaceae)	0.353	167928
13	14.15	Wogonin	*Scutellaria baicalensis* Georgi (Lamiaceae)	0.0503	5281703
14	14.20	Rhein	*Rheum officinale* Baill. (Polygonaceae)	2.72	10168
15	14.31	Saikosaponin D	*Bupleurum chinensis* DC. (Apiaceae)	0.386	107793
16	14.33	Emodin	*Rheum officinale* Baill. (Polygonaceae)	0.0742	3220
17	14.43	Morusin	*Morus alba var. tatarica* (L.) Loudon (Moraceae)	0.0625	5281671

Supplementary Table 3. Primers for qRT-PCR.

Gene name	Forward primer (5'-3')	Reverse primer (5'-3')
Muc2	ATGCCCACCTCCTCAAAGAC	TGCCGTGTGGATACAGGATG
Claudin-1	GGGGACAACATCGTGACCG	AGGAGTCGAAGACTTTGCACT
Occludin	TTGAAAGTCCACCTCCTTACAGA	CCGGATAAAAAGAGTACGCTGG
ZO1	GCCGCTAAGAGCACAGCAA	TCCCCACTCTGAAAATGAGGA
Ifnb1	AGCTCCAAGAAAGGACGAACA	GCCCTGTAGGTGAGGTTGAT
M1	GCATCGGTCTCATAGGCAAATG	CCTCTGCTGCTTGCTCACTC
NS1	GCGATGCCCCATTCCTTG	ATCCGCTCCACTATCTGCTTTC
Ifnb1	ATGACCAACAAGTGTCTCCTCC	GGAATCCAAGCAAGTTGTAGCTC
GAPDH	CTGGGCTACACTGAGCACC	AAGTGGTCGTTGAGGGCAATG
β-Actin	AGTGTGACGTTGACATCCGT	GCAGCTCAGTAACAGTCCGC

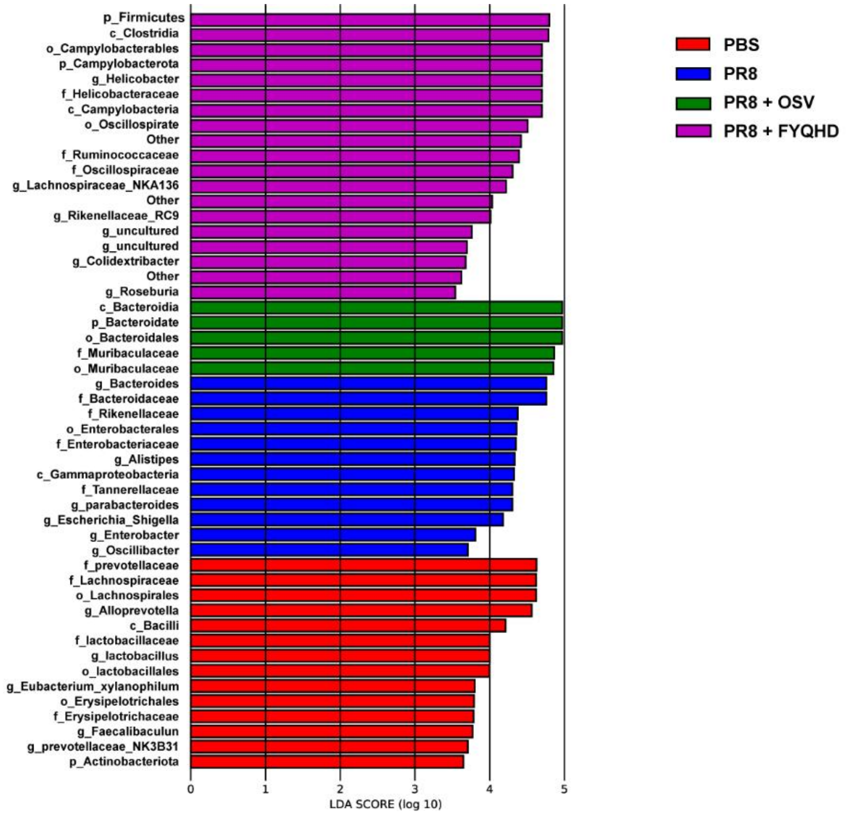

Supplementary Figure 3. Linear discriminant analysis (LDA) effect size (LEfSe) analysis was used to detect differences in the flora between the 4 groups (LDA >3).

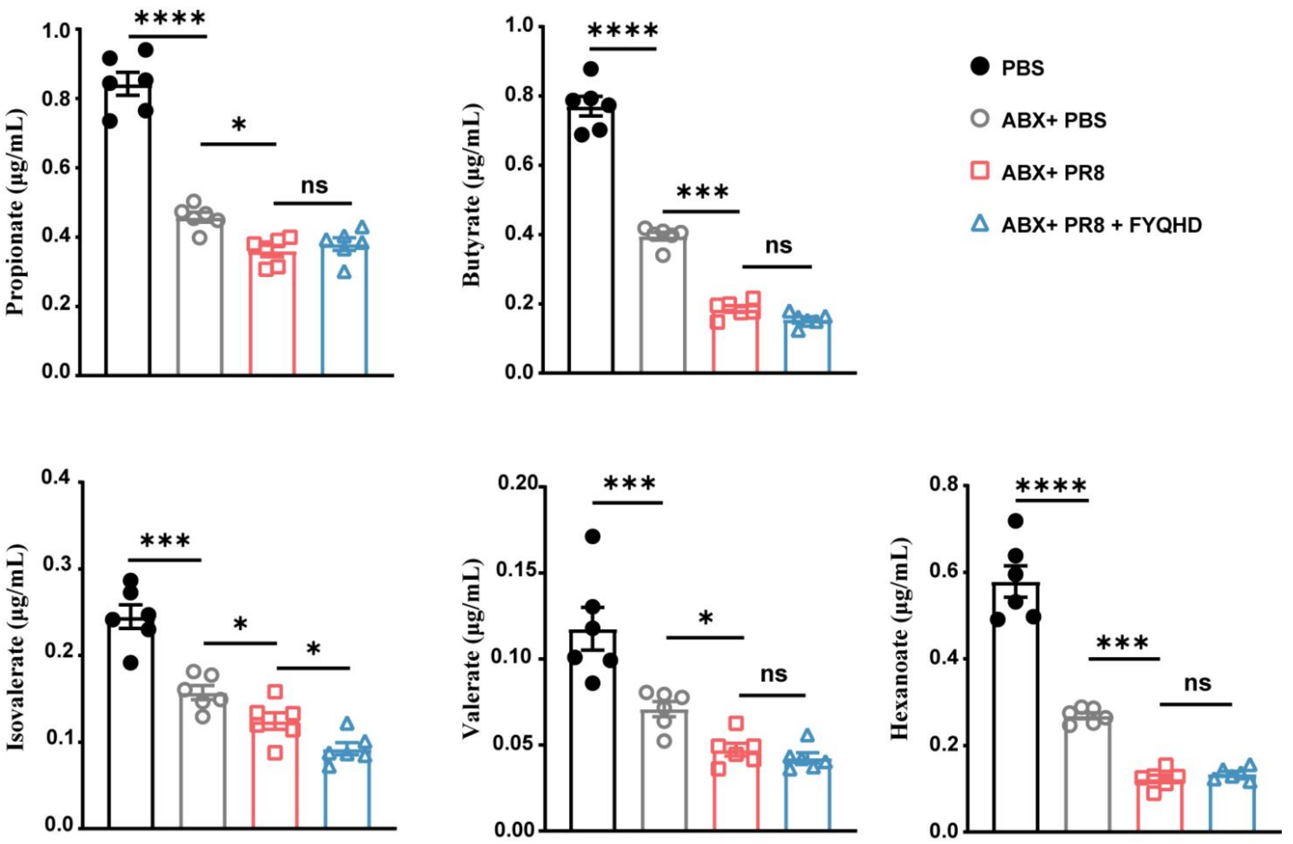

Supplementary Figure 4. Levels of SCFAs (propionate, butyrate, isovalerate, valerate, hexanoate) in mice treated with ABX. Statistical data were displayed with mean ± SD (n = 6 mice per group) and analysed using one-way analyses of variance (ANOVA) followed by Tukey's post hoc analysis. ns, not significant, $^{*}P < 0.05$, $^{***}P < 0.001$, $^{****}P < 0.0001$ *vs.* ABX + PR8 group.

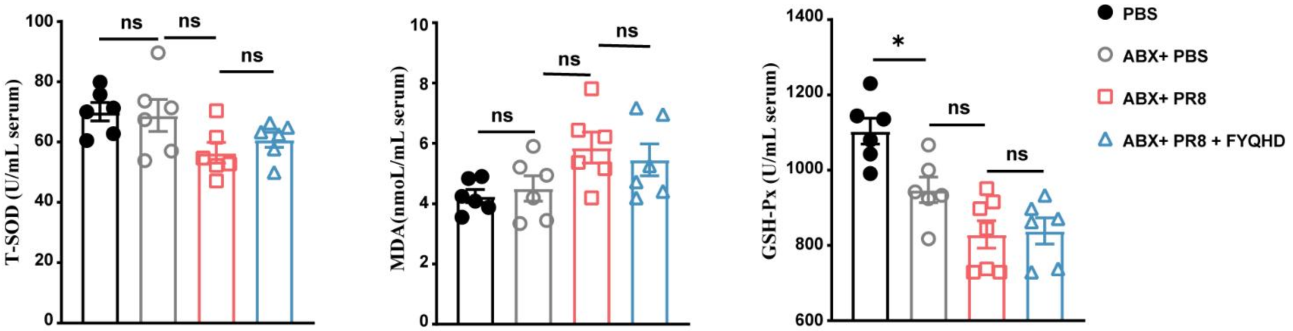

Supplementary Figure 5. Concentrations of T-SOD, MDA and GSH-Px in sera from each group (n = 6). Statistical data were displayed with mean ± SD (n = 6 mice per group) and analysed using one-way analyses of variance (ANOVA) followed by Tukey's post hoc analysis. ns, not significant, $^{*}P < 0.05$ *vs.* ABX + PR8 group.

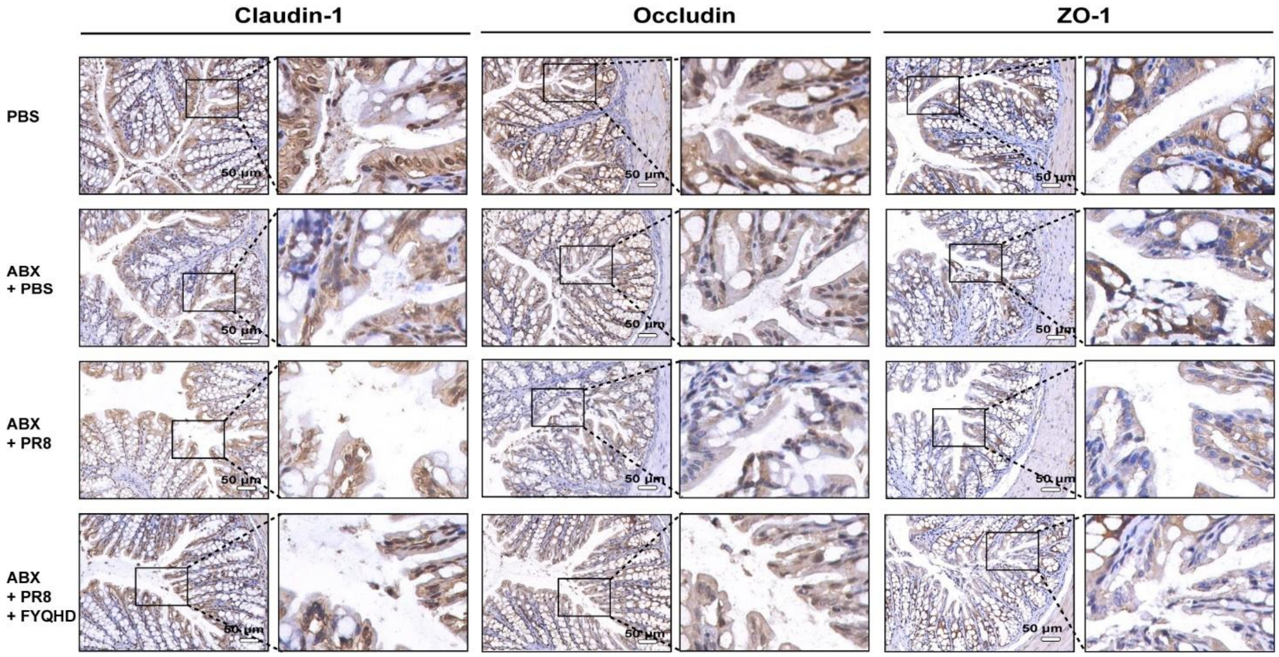

Supplementary Figure 6. Immunohistochemical staining of Occludin, Claudin-1 and ZO-1 in colons of different groups. (Scale bar: 50 μm).

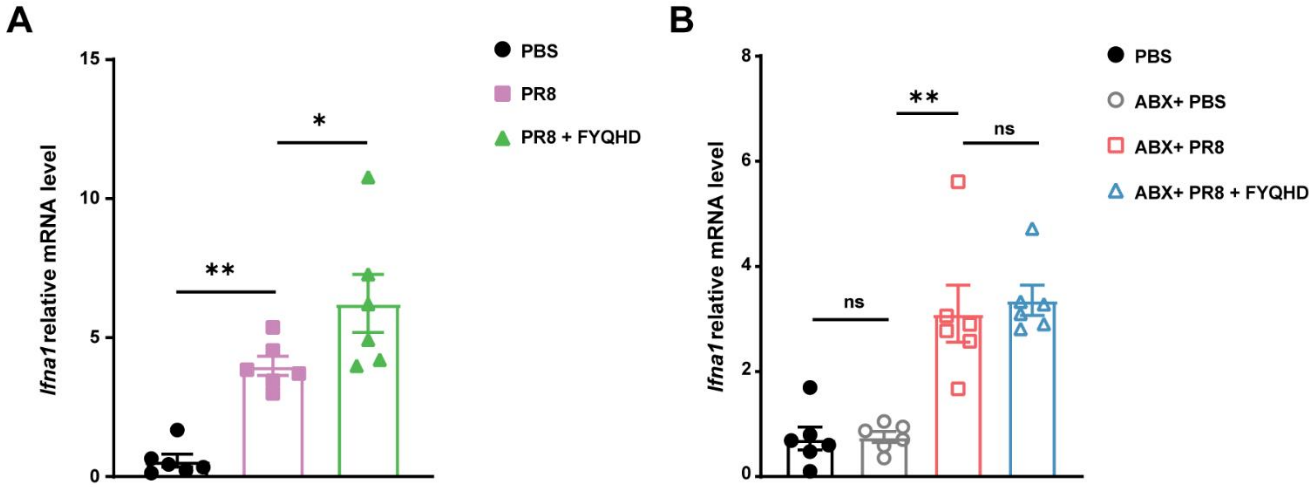

Supplementary Figure 7. The expression of *Ifna1* detected in lung tissues by real-time PCR on day 7 post-infection (n = 6). Data were presented as means ± SEM. Statistical significance was determined using one-way ANOVA, followed by Tukey test. ns, not significant, $*P < 0.05$, $**P < 0.01$ *vs.* PR8 group or *vs.* ABX + PR8.
